# Alkylator-Induced and Patient-Derived Xenograft Mouse Models of Therapy-Related Myeloid Neoplasms Model Clinical Disease and Suggest the Presence of Multiple Cell Subpopulations with Leukemia Stem Cell Activity

**DOI:** 10.1371/journal.pone.0159189

**Published:** 2016-07-18

**Authors:** Brian A. Jonas, Carl Johnson, Dita Gratzinger, Ravindra Majeti

**Affiliations:** 1 Department of Internal Medicine, Division of Hematology and Oncology, University of California Davis Comprehensive Cancer Center, University of California Davis School of Medicine, Sacramento, CA, United States of America; 2 Department of Medicine, Division of Hematology, Cancer Institute, and Institute for Stem Cell Biology and Regenerative Medicine, Stanford University School of Medicine, Stanford, CA, United States of America; 3 Department of Pathology, Stanford University School of Medicine, Stanford, CA, United States of America; B.C. Cancer Agency, CANADA

## Abstract

Acute myeloid leukemia (AML) is a heterogeneous group of aggressive bone marrow cancers arising from transformed hematopoietic stem and progenitor cells (HSPC). Therapy-related AML and MDS (t-AML/MDS) comprise a subset of AML cases occurring after exposure to alkylating chemotherapy and/or radiation and are associated with a very poor prognosis. Less is known about the pathogenesis and disease-initiating/leukemia stem cell (LSC) subpopulations of t-AML/MDS compared to their *de novo* counterparts. Here, we report the development of mouse models of t-AML/MDS. First, we modeled alkylator-induced t-AML/MDS by exposing wild type adult mice to N-ethyl-N-nitrosurea (ENU), resulting in several models of AML and MDS that have clinical and pathologic characteristics consistent with human t-AML/MDS including cytopenia, myelodysplasia, and shortened overall survival. These models were limited by their inability to transplant clinically aggressive disease. Second, we established three patient-derived xenograft models of human t-AML. These models led to rapidly fatal disease in recipient immunodeficient xenografted mice. LSC activity was identified in multiple HSPC subpopulations suggesting there is no canonical LSC immunophenotype in human t-AML. Overall, we report several new t-AML/MDS mouse models that could potentially be used to further define disease pathogenesis and test novel therapeutics.

## Introduction

Acute myeloid leukemia (AML) is an aggressive bone marrow malignancy characterized by the accumulation of immature myeloid cells with defective maturation and function. AML is a heterogeneous disease and is classified by the World Health Organization into several subtypes on the basis of cytogenetic, molecular, and phenotypic characteristics [1]. Therapy-related myeloid neoplasms (t-MNs), consisting of therapy-related AML (t-AML) and therapy-related myelodysplastic syndrome (t-MDS), are one such subtype accounting for 10–20% of AML cases and occur in patients previously treated with radiation and/or chemotherapy for other diseases [2]. t-AML/MDS is typically diagnosed 5–7 years after previous treatment, and the t-AML phase can be preceded by a t-MDS phase characterized by cytopenias related to bone marrow failure and less than 20% bone marrow blasts [3, 4]. t-AML/MDS is clinically characterized by deletions in chromosomes 5 and/or 7 in nearly 70% of cases and by a distinct set of recurrent molecular mutations, including TP53 [3, 5–8]. TP53 mutations are likely an early event in the pathogenesis of these diseases [6, 9, 10]. While *de novo* AML is associated with a 30–40% 5-year overall survival (OS) with current standard therapies, t-AML/MDS has an even worse prognosis, with a 5-year OS of less than 10% [3, 4].

A growing body of evidence indicates that *de novo* AML is composed of a cellular hierarchy initiated and maintained by self-renewing leukemia stem cells (LSC) that are functionally defined by their ability to reconstitute AML in xenograft models [11]. The cellular hierarchy in AML is analogous to normal hematopoiesis in which multipotent, self-renewing hematopoietic stem cells (HSC) give rise to downstream progenitor cells and ultimately all mature blood elements [12]. Recent work has demonstrated that the disease stem cells in MDS are found in the HSC compartment [13–17]. Several lines of evidence argue that AML and MDS arise from the stepwise accumulation of multiple mutations in pre-leukemic HSC, eventually generating LSC capable of initiating disease [18–20]. One prediction of the LSC model is that relapse is common in AML and MDS because the mostly quiescent LSC are not eliminated by conventional therapies that preferentially target rapidly dividing cells, such as downstream leukemic progenitor cells and blasts [21]. The clinical significance of the LSC model in AML has been confirmed by studies showing that presence of a LSC gene expression signature is associated with inferior clinical outcomes [22, 23].

Numerous mouse models of AML and MDS have been described in order to improve understanding of disease pathogenesis and test novel therapeutic approaches [24–28]. Xenograft models in immunocompromised mice were used to develop the AML LSC model, with CD34+CD38- emerging as the canonical immunophenotype of AML and MDS stem cells [21, 29, 30]. Additional studies have also demonstrated LSC activity in other immunophenotypic cell subpopulations, including CD34+CD38+ and CD34- cells [23, 31–34]. Importantly, previous studies have not specifically defined LSC in t-AML/MDS. A number of other investigators have used genetic approaches to model AML and MDS in mice, including NF1, RUNX1, FLT3-ITD, NPM1, and DICER [35–38]. In some cases, these models have been used to describe secondary events leading to AML after treatment of animals with alkylators or radiation [35, 39–41]. However, mouse models relying on transgenic or virally transduced expression of oncogenes might not accurately model clonal mutation accumulation in human t-AML/MDS induced by the nonspecific genetic damage caused by alkylating chemotherapy and radiation therapy. A few studies have described generation of alkylator-induced or radiation-induced AML by exposure of wild type mice to ENU or ionizing radiation, and it is possible such approaches might better model human t-MNs [42–45].

Here, we report the development of new mouse models of t-AML/MDS using both wild type mice and human patient samples. We treated wild type adult mice with ENU and monitored for development of t-AML/MDS. Moribund mice were diagnosed with AML, MDS, or other diseases through extensive pathologic characterization and in accordance with guidelines from the Mouse Models of Human Cancers Consortium (MMHCC) [46, 47]. In parallel, we also established xenograft models from primary human samples of t-AML and used these models to evaluate human t-AML cell subpopulations for LSC activity.

## Materials and Methods

### Animal Care

All mouse experiments were carried out in strict accordance with the recommendations in the *Guide for the Care and Use of Laboratory Animals* from the National Institutes of Health and followed protocols approved by the Stanford University Institutional Animal Care and Use Committee (Protocol # 22264). Mice were housed in sterile cages with sterile bedding and subjected to daily light/dark cycles. There were no more than five mice per cage. Mice were maintained on sterile water and chow containing trimethoprim-sulfamethoxazole. Mice were inspected daily by the veterinary staff. Fifty wild type 9–12 week-old DBA/2J (n = 40) or SWR/J mice (n = 10) (The Jackson Laboratory, Bar Harbor, ME, USA) were treated with two weekly intraperitoneal doses of N-ethyl-N-nitrosurea (ENU, Sigma-Aldrich, St. Louis, MO, USA) totaling 100–300 mg/kg per animal. For all human and mouse transplantation experiments, recipient 8–12 week old NOD/SCID/IL2R-gamma-null (NSG, The Jackson Laboratory) mice were conditioned with sublethal X-irradiation (200 rads) followed by tail vein injections containing donor cells. All mice were observed with at least weekly physical exams for signs and symptoms of disease and with monthly weight checks, complete blood count (CBC) using a HemaTrue blood analyzer (Heska, Loveland, CO, USA), peripheral blood (PB) smear, and flow cytometric analysis of PB cell composition. Mice that exhibited ruffled fur, hunched posture, weight loss or severe CBC abnormalities were considered moribund and were euthanized using carbon dioxide asphyxiation. Anesthetization with isofluorane inhalation, in addition to carprofen and normal saline injections, were used to minimize pain associated with experimental procedures.

### Human Samples

All human AML samples were obtained from patients at the Stanford Medical Center with written informed consent in accordance with the Stanford University Institutional Review Board (IRB) approved protocols (Stanford IRB numbers 18329 and 6453). Mononuclear cells from each sample were isolated by Ficoll separation and cryopreserved in liquid nitrogen. All experiments used freshly thawed cells.

### Mouse Necropsy and Cell Suspension Preparation

Mice were sacrificed when moribund and necropsy performed to identify any gross pathologic abnormalities and to measure the spleen size. Cell suspensions were then made from the PB, bone marrow (BM), spleen, and any tumors. PB mononuclear cells were obtained from mouse whole blood through a two-step purification process using 2% Dextran followed by incubation with Ammonium-Chloride-Potassium (ACK) Lysing Buffer (Gibco, Grand Island, NY, USA). BM cells were isolated from the skeleton using a mortar and pestle. BM, spleen, and any tumors were passed through a 100 micron nylon cell strainer (Corning Life Sciences, Tewksbury, MA, USA) to create single cell suspensions which were then incubated with ACK Lysing Buffer to deplete red blood cells. Mononuclear cells from each sample were isolated by Histopaque (Sigma-Aldrich) separation, analyzed, and cryopreserved in liquid nitrogen. PB smears were also prepared from each mouse at the time of sacrifice. In addition, smears of BM, spleen, liver, and any tumors were prepared by using a 5/0 paintbrush dipped in hypotonic fetal bovine serum to brush cells onto microscope slides. PB and paintbrush smears were fixed and stained using the Harleco Hemacolor staining system (Merck KGaA, Darmstadt, Germany). In addition, tissue samples from BM, spleen, liver, kidney, lungs, and brain were fixed in 10% formalin. These samples were sent to HISTO-TEC Laboratory (Hayward, CA, USA) where they were decalcified, paraffin-embedded, sectioned, and either stained with hematoxylin and eosin or immunostained with MPO or CD3. All slides were evaluated and images obtained using a Leica DM5500B upright microscope and camera system (Leica Microsystems, Buffalo Grove, IL, USA). All diagnoses were made according to guidelines from the MMHCC [46, 47] and were verified by a hematopathologist (DG).

### Flow Cytometry Analysis and Cell Sorting

The following anti-mouse monoclonal antibodies were used: Ter119 PE-Cy5 (clone TER-119, rat host), CD45.1 PE-Cy7 (A20, mouse), c-Kit APC-Cy7 and PE-Cy7 (2B8, rat), CD34 FITC (RAM34, rat), CD16/32 A700 (93, rat), Gr-1 PE-Cy5 (RB6-8C5, rat), Mac-1 PE-Cy5 (M1/70, rat), CD42d PE (1C2, Armenian hamster), CD71 FITC (R17217, rat), IL-7Ra A700 and PE-Cy5 (A7R34, rat), B220 APC and PE-Cy5 (RA3-6B2, rat), CD19 PE and PE-Cy5 (eBio1D3, rat), CD43 FITC (eBioR2/60, rat), TCRb APC (H57-597, Armenian hamster), CD3e PE, PE-Cy5 and eFluor450 (145-2C11, Armenian hamster), CD4 PE-Cy5 and PE-Cy7 (GK1.5, rat), CD8a FITC and PE-Cy5 (53–6.7, rat), and CD5 PE (53–7.3, rat) from eBioscience (San Diego, CA, USA), and CD45.2 AF488 and BV510 (104, mouse), Sca-1 PB (D7, rat), Flk-2 APC (A2F10, rat), Gr-1 APC (RB6-8C5, rat), Mac-1 PB (M1/70, rat), F4/80 A700 (BM8, rat), and IgM PE-Cy7 (RMM-1, rat) from Biolegend (San Diego, CA, USA). The following mouse stains were used: hematopoietic stem and progenitor (HSPC)–Lineage Cocktail (Ter119, Mac-1, Gr-1, B220, CD19, CD3e, CD4, CD8a, and IL-7Ra, all 1:300), c-Kit (1:50), Sca-1 (1:50), CD34 (1:25), CD16/32 (1;50), and Flk-2 (1:50); Myeloid–Ter119, CD45.2, c-Kit, Gr-1, Mac-1, F4/80, CD42d, and CD71 (all 1:50); B-lymphoid–Ter119, CD45.2, IL-7Ra, IgM, B220, CD19, and CD43 (all 1:50); T-lymphoid–Ter119, CD45.2, TCRb, CD3e, CD4, CD8a, and CD5 (all 1:50); and Engraftment lineage–Ter119, CD45.1, CD45.2, c-Kit, Gr-1, Mac-1, B220, and CD3e (all 1:50). The following anti-human monoclonal antibodies were used: CD45 V450 (HI30, mouse), CD34 APC (8G12, mouse), CD38 PE-Cy7 (HB7, mouse), CD19 PE-Cy5 and APC (HIB19, mouse), CD20 PE-Cy5 (2H7, mouse), CD99 FITC (TU12, mouse), CD3 APC-Cy7 (SK7, mouse), and CD33 PE (WM53, mouse) from BD Biosciences (San Jose, CA, USA), and Tim3 PE (F38-2E2, mouse) from eBioscience. The following human stains were used: HSPC–CD34 (1:50), CD38 (1:100), Tim3 (1:5), CD99 (1:5), CD3 (1:25), CD19 (1:50), and CD20 (1:50); and Engraftment–mTer119 (1:200), mCD45.1 (1:200), CD45 (1:100), CD3 (1:50), CD19 (1:100), and CD33 (1:100). Propidium iodide (1:1000) was used to exclude dead cells. Cells were suspended in FACS buffer (phosphate-buffered saline with 2% FBS and 2mM ethylenediaminetetraacetic acid) and incubated (30-90mins at 4°C) with the appropriate antibody stains. Fluorescence activated cell sorter (FACS) analysis and sorting was performed using a BD FACS Aria II flow cytometer (BD Biosciences).

### NSG Transplantation Assays

Bulk BM cells, FACS CD3-depleted BM cells, or FACS purified BM subpopulations from ENU-induced mouse diseases were transplanted into NSG recipients. Human therapy-related AML samples were CD3-depleted using Robosep (Stem Cell Technologies, Vancouver, BC, Canada) before undergoing FACS purification of leukemia cell subpopulations and transplantation into NSG recipients. NSG mice were conditioned, injected, and monitored as previously described.

### Data Analysis

Flow cytometry data were analyzed using FlowJo software (TreeStar Inc., Ashland, OR). Figures and statistical analysis were performed using Prism (GraphPad Software, Inc., La Jolla, CA).

## Results

### Treatment of wild type mice with ENU leads to development of mouse AML and MDS with clinical and pathologic features of human disease

A total of fifty 9–12 week old adult mice were administered two weekly intraperitoneal doses of the alkylating agent, ENU. Ten SWR/J and 20 DBA/2J mice received 300 mg/kg, 10 DBA/2J received 200 mg/kg, and 10 DBA/2J received 100 mg/kg ENU. Mice were monitored with weekly physical examinations and monthly evaluations including weight check, CBC, peripheral blood (PB) smears, and PB flow cytometric analysis. Mice were sacrificed when moribund and underwent necropsy, spleen size assessment, and flow cytometric analysis of PB and bone marrow (BM) cell compositions. Smears were prepared from the PB, BM, spleen, liver, and any tumors or enlarged lymph nodes for morphologic analysis. In addition, formalin-fixed paraffin-embedded (FFPE) sections of BM, spleen, liver, kidney, lungs, and brain were stained with H&E. Diagnoses were determined by evaluation of these clinical and pathologic assessments in accordance with guidelines from the MMHCC. Key defining criteria for a diagnosis of AML included: (1) Diffuse involvement of hematopoietic tissues; (2) Cytopenia(s); (3) Increased nonlymphoid hematopoietic cells in the spleen; (4) Neoplastic cells in non-hematopoietic tissues or leukocytosis with at least 20% immature forms/blasts; and (5) Malignant behavior including at least 20% immature forms/blasts in the blood, spleen, or bone marrow, rapidly fatal to the primary animal, or transplantable and rapidly fatal (within eight weeks) to secondary recipients [47]. Key diagnostic criteria for MDS included: (1) Cytopenia(s); (2) Dysplastic hematopoiesis or at least 20% immature forms/blasts; and (3) Does not meet criteria for AML [47]. The final diagnosis was given to each primary mouse case after independent review of the pathologic specimens by a hematopathologist blinded to the clinical data.

**Fig 1:**
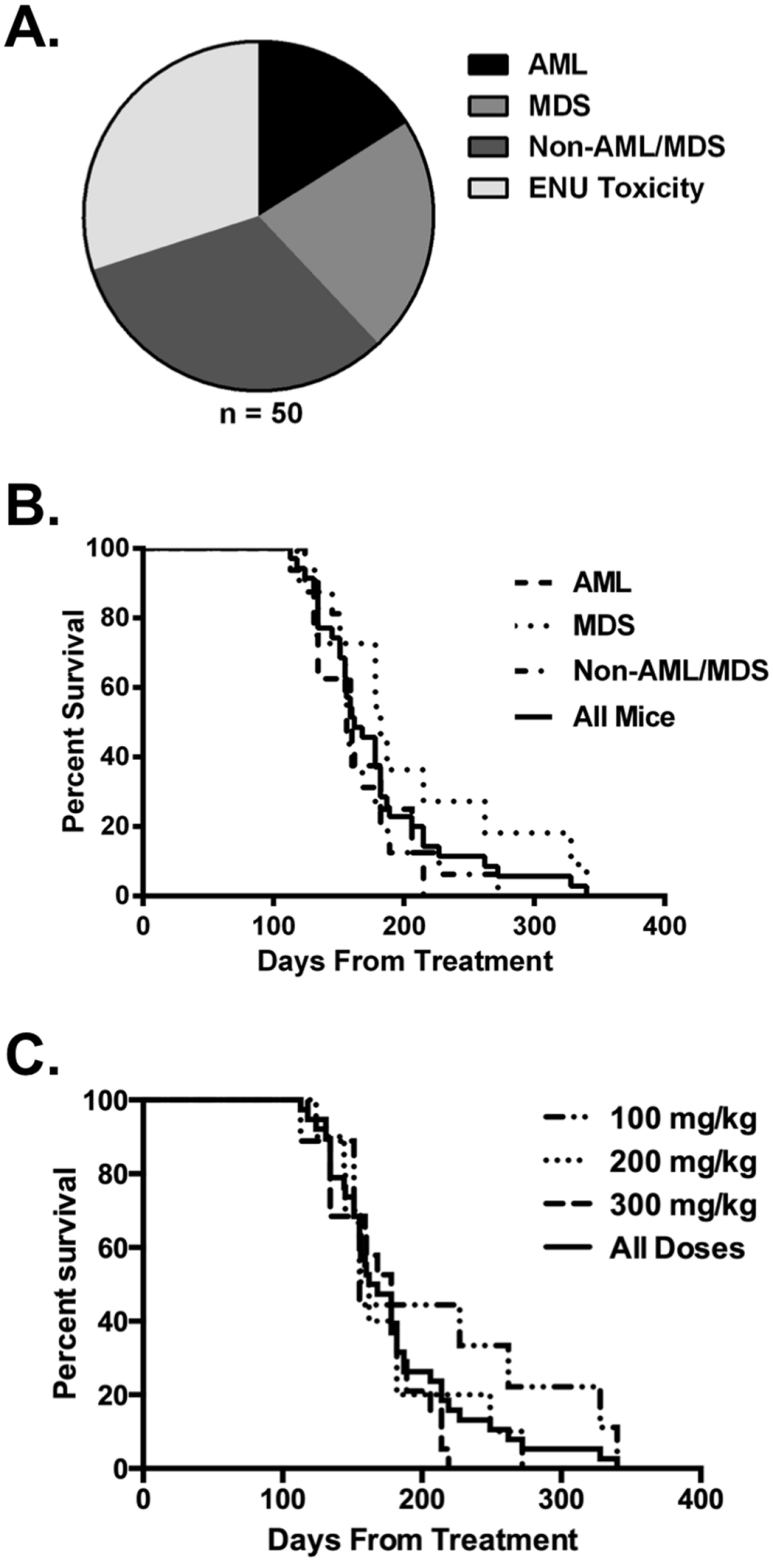
Treatment of mice with ENU leads to development of therapy-related myeloid neoplasms. **(a)** Forty DBA/2J and ten SWR/J mice were treated with 100–300 mg/kg ENU, monitored for disease, and sacrificed when moribund. Diagnostic analysis revealed AML, MDS, and non-AML/MDS diseases in 16%, 22%, and 32% of mice respectively. The remaining 30% of mice died from ENU-toxicity or were not analyzable. **(b)** Kaplan-Meier survival analysis of ENU-treated (n = 35) mice shows a median overall survival of 162 days from the date of treatment. There were no statistically significant differences in survival by diagnosis (n = 8, n = 11, and n = 16 for AML, MDS, and non-AML/MDS diseases respectively). **(c)** Kaplan-Meier survival analysis of ENU-treated mice shows no significant difference in survival among the various ENU dose levels tested (n = 35, n = 9, n = 10, n = 16 for total, 100 mg/kg, 200 mg/kg, and 300 mg/kg respectively).

Out of a total of 50 ENU-treated mice, 8 mice were diagnosed with AML, 11 mice with MDS, and 16 mice with non-AML/MDS diseases (Fig 1A and Table A in S1 File). Non-AML/MDS diseases included T-lymphoblastic leukemia, B-lymphoblastic leukemia, myeloproliferative neoplasm, mast cell sarcoma, and carcinoma. Three mice with MDS had a concurrent carcinoma of unclear significance. There was no involvement of the bone marrow by carcinoma in these mice. Fifteen mice died early from complications of the ENU-treatment or were not analyzable and are not included in subsequent analyses (Table A in S1 File). The 300 mg/kg ENU dose level was most strongly associated with development of myeloid disease (13 of 19 AML/MDS cases), but was also associated with the most toxicity as all 12 early ENU-related deaths were at the 300 mg/kg dose level. There were no significant differences between SWR/J and DBA/2J mice treated at the 300 mg/kg ENU dose level, with 10% AML, 40% MDS, 40% ENU toxicity and 10% non-AML/MDS, and 25% AML, 20% MDS, 40% ENU toxicity and 15% non-AML/MDS, respectively.

Survival from the day of the first ENU injection was determined for each mouse diagnosed with a myeloid or non-AML/MDS disease. Kaplan-Meier survival analysis was then applied to determine median overall survival (OS) of the entire cohort of animals, excluding mice that were not evaluable due to direct ENU toxicity or unanticipated early death. Median OS for the 35 mice diagnosed with AML, MDS, or non-AML/MDS diseases was 162 days from the day of first ENU treatment (Fig 1B and 1C, solid line). There were no significant differences in survival when mice were stratified by diagnosis (Fig 1B) or ENU dose (Fig 1C).

**Fig 2:**
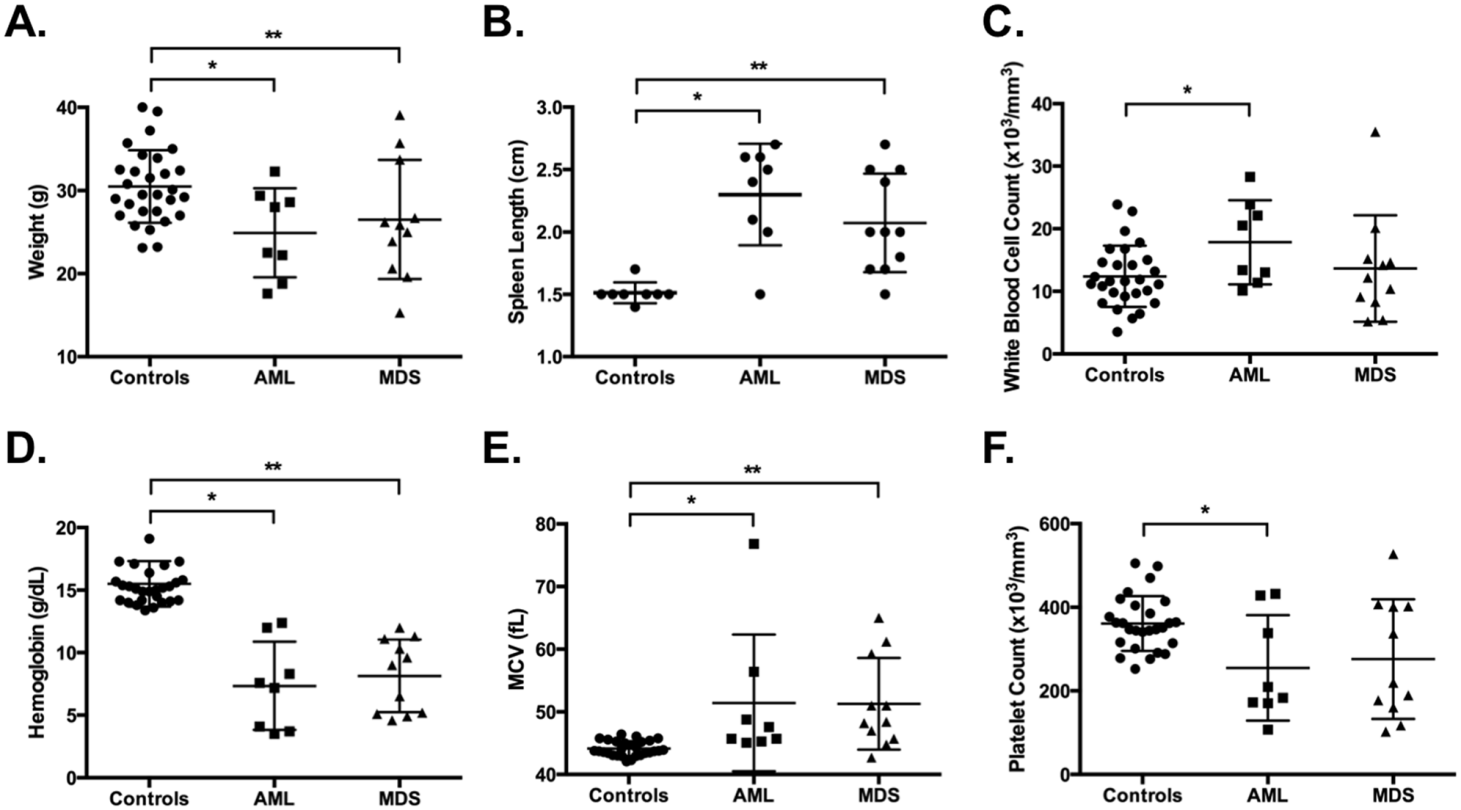
Mice with AML and MDS exhibit weight loss, splenomegaly, and blood count abnormalities at the time of sacrifice. Scatter plots with means and standard deviations for controls [n = 29, except for **(b)** where n = 8], AML (n = 8), and MDS (n = 11) are shown for: weight in g **(a)**; spleen length in cm **(b)**; WBC in 10^3^ cells/mm^3^ **(c)**; Hgb in g/dL **(d)**; MCV in fL **(e)**; and platelet count in 10^3^ platelets/mm^3^ **(f)**. * p<0.05 for controls versus AML by Mann-Whitney test; ** p<0.05 for controls versus MDS by Mann-Whitney test.

Clinical features, including weight, spleen length, and CBC were collected for each diseased mouse (AML, n = 8 and MDS, n = 11) at the time of sacrifice and are summarized in Fig 2 and Table A in S1 File. Untreated, age-matched cohorts of DBA/2J and SWR/J mice (n = 29 total) served as controls. Spleen length was determined in eight of these age-matched controls. The mean weight was significantly lower in AML and MDS mice compared to controls (Fig 2A, p<0.05). Spleen size, measured as mean spleen length, was markedly increased in AML and MDS mice compared to controls (Fig 2B, p<0.05). Leukocytosis was seen in AML cases with a mean WBC significantly higher than control (Fig 2C, p<0.05). Mean hemoglobin was significantly lower and average mean corpuscular value (MCV) higher in both AML and MDS cases relative to control (Fig 2D and 2E, p<0.05). Platelet counts were variable overall among the disease cases, but were significantly lower in AML cases compared to controls (Fig 2F, p<0.05). Overall, the clinical features of ENU-induced AML and MDS cases were consistent with human AML and MDS.

**Fig 3:**
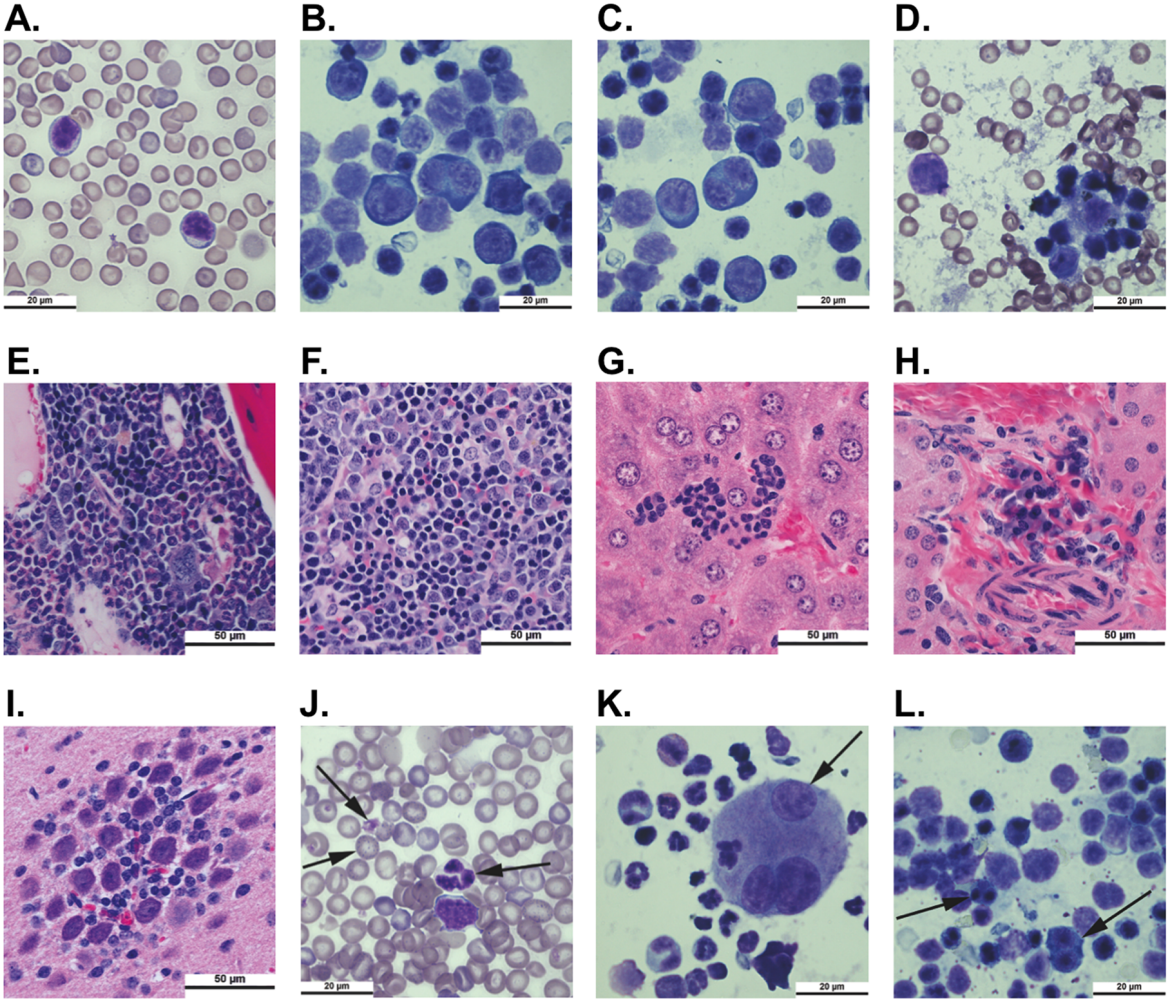
ENU-treated mice demonstrate morphologic changes consistent with AML or MDS. Smears from peripheral blood, bone marrow, spleen, and liver were prepared from mice at sacrifice and stained with Wright-Giemsa stain. Samples of tibia, spleen, liver, kidney, and brain were fixed in formalin, paraffin-embedded, sectioned, and stained with hematoxylin and eosin. Representative smears (1000x) from an AML case show circulating peripheral blood blasts **(a)** and blasts infiltrating the bone marrow **(b)**, spleen **(c)**, and liver **(d).** Representative images (400x) show infiltrates of blasts in bone marrow **(e)**, spleen **(f)**, liver **(g)**, kidney **(h)**, and brain **(i)** sections from a mouse with AML. Representative smears (1000x) from the peripheral blood **(j)**, bone marrow **(k)**, and spleen **(l)** from a mouse with MDS show dysplastic hematopoiesis including abnormal neutrophil nuclear maturation, target cells, basophilic stippling, and large platelets in the peripheral blood, abnormal megakaryocyte nuclear morphology in the bone marrow, and bi-nucleated red cell precursors in the spleen. Examples are highlighted with arrows.

Furthermore, the ENU-induced mouse AML and MDS cases had morphologic and histologic features of human AML and MDS and fulfilled the diagnostic criteria established by the MMHCC. Wright-Giemsa staining of PB, BM, spleen, and liver smears revealed circulating blasts and infiltrating blast populations in AML cases. Representative images are shown in Fig 3A–3D. Similarly, H&E sections from various FFPE tissues, including BM, spleen, liver, kidney, and brain, demonstrated infiltration by blasts in AML cases. Representative images are shown in Fig 3E–3I. Dysplasia in myeloid, erythroid, and/or megakaryocyte lineages was seen in smears from PB, BM, and spleen in MDS cases. Dysgranulopoietic changes most commonly were manifested by open chromatin in mature cells and lobated nuclei (instead of the normal ring-shaped structure of mouse neutrophils). Notable dyserythropoietic findings included macrocytosis, target cells, basophilic stippling, and erythroblasts with multiple nuclei and nuclear fragmentation. For the megakaryocyte lineage, dysplastic changes observed included large and hypogranular platelets, micromegakaryocytes, and megakaryocytes with multiple separated nuclei. Representative images of these findings are shown in Fig 3J–3L.

### Mice with ENU-induced AML and MDS have altered bone marrow cell composition

**Fig 4:**
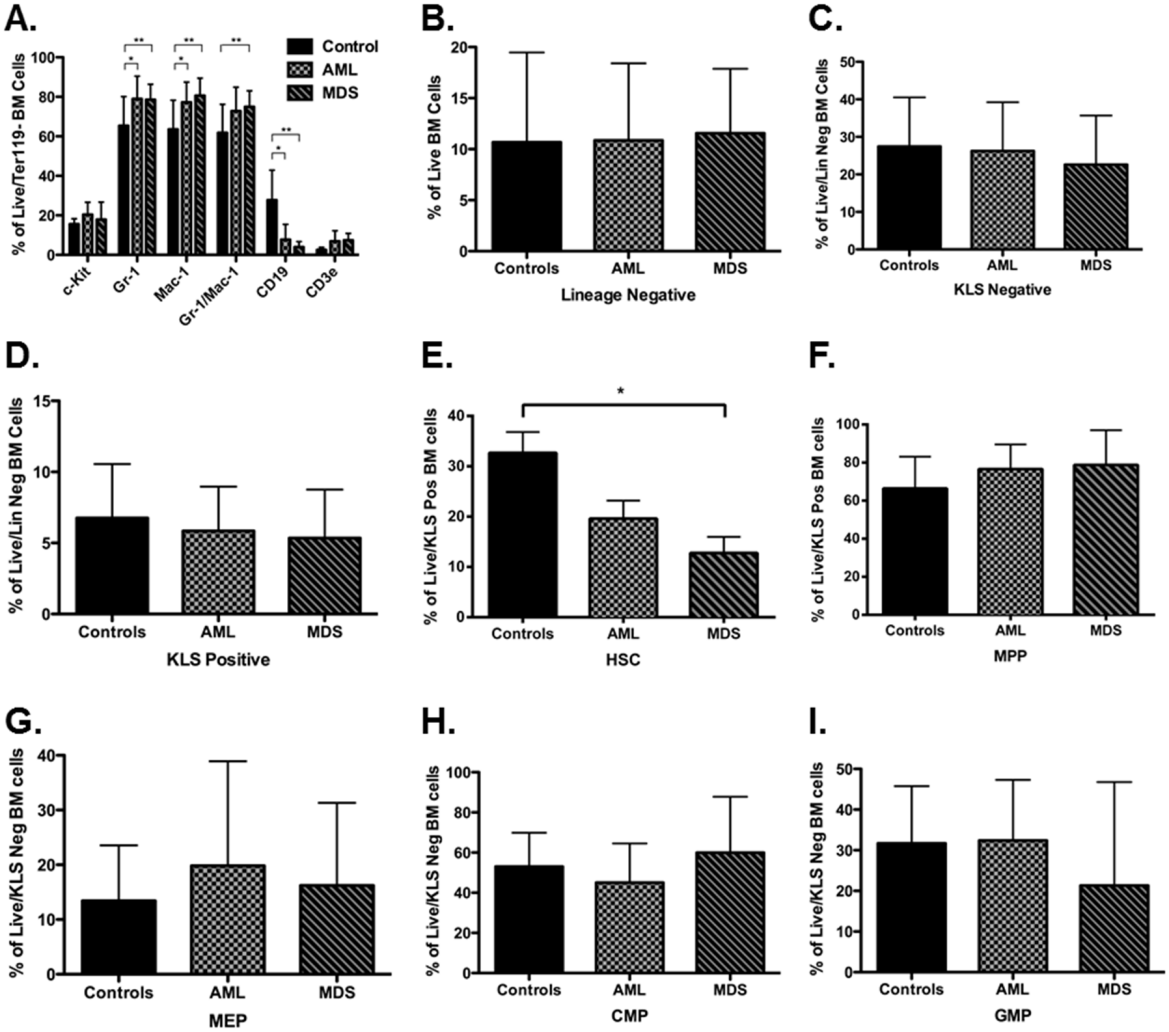
Mice with AML and MDS show immunophenotypic changes in bone marrow cell composition compared to untreated age-matched control mice. Single-cell suspensions of bone marrow cells were prepared at the time of sacrifice and analyzed for relative expression of myeloid, lymphoid, and hematopoietic progenitor cell markers by flow cytometry. **(a)** Expression of myeloid and lymphoid markers in control (n = 6), AML (n = 9), and MDS (n = 8) mice. Mean percentages of live/Ter119 negative BM cells are shown with standard deviations. Mice with AML and MDS have increased frequency of myeloid cells (Gr-1+ and Mac-1+) and decreased frequency of B-lymphocyte cells (CD19+) compared to control mice (* p<0.05 for controls versus AML by 2-way ANOVA test; ** p<0.05 for controls versus MDS by 2-way ANOVA test). **(b-i)** Immunophenotypic analyses of bone marrow hematopoietic precursor populations in control (n = 16), AML (n = 8), and MDS (n = 8) mice are shown. Mean percentages with standard deviations of live **(b)**, live/lineage negative **(c, d)**, live/KLS positive **(e-f),** and live/KLS negative **(g-i)** BM cells are shown. **(e)** HSC were significantly decreased in MDS mice, but not AML mice, compared to controls (* p<0.05 by Mann-Whitney test). There were no other significant changes in hematopoietic precursor cell percentages, including lineage negative **(b)**, KLS negative **(c)**, KLS positive **(d)**, MPP **(f)**, MEP **(g)**, CMP **(h)**, and GMP **(i)**.

To complete the clinical and pathologic characterization of ENU-induced AML and MDS cases, the relative compositions of BM lineage positive and HSPC subpopulations were determined by flow cytometry and compared to age-matched controls. Among lineage positive cell subpopulations, myeloid cells (Gr-1+ and Mac-1+) were increased and B-lymphoid cells (CD19+) decreased in AML and MDS mice compared to control mice (Fig 4A, p<0.05). There were no significant differences in c-Kit+ or CD3+ cells. Among the various HSPC subpopulations, only HSC (c-Kit+/LineageNegative/Sca-1+/CD34-/Flk-2-) were significantly decreased in MDS, but not AML, mice compared to control (Fig 4E, p<0.05). There were no significant differences in lineage negative (Lin-, Fig 4B), c-Kit+/Lin-/Sca-1- (KLS negative, Fig 4C), c-Kit+/Lin-/Sca-1+ (KLS positive, Fig 4E), multipotent progenitors (MPP, KLS positive/CD34+/Flk-2lo-pos, Fig 4F), megakaryocyte-erythroid progenitors (MEP, KLS negative/CD34-/FcgR3-, Fig 4G), common myeloid progenitors (CMP, KLS negative/CD34-/FcgR3lo, Fig 4H), or granulocyte-monocyte progenitors (GMP, KLS negative/CD34-/FcgR3+, Fig 4I) in AML or MDS mice compared to controls.

### ENU-induced AML and MDS models are transplantable but do not lead to lethal disease in recipient mice

**Fig 5:**
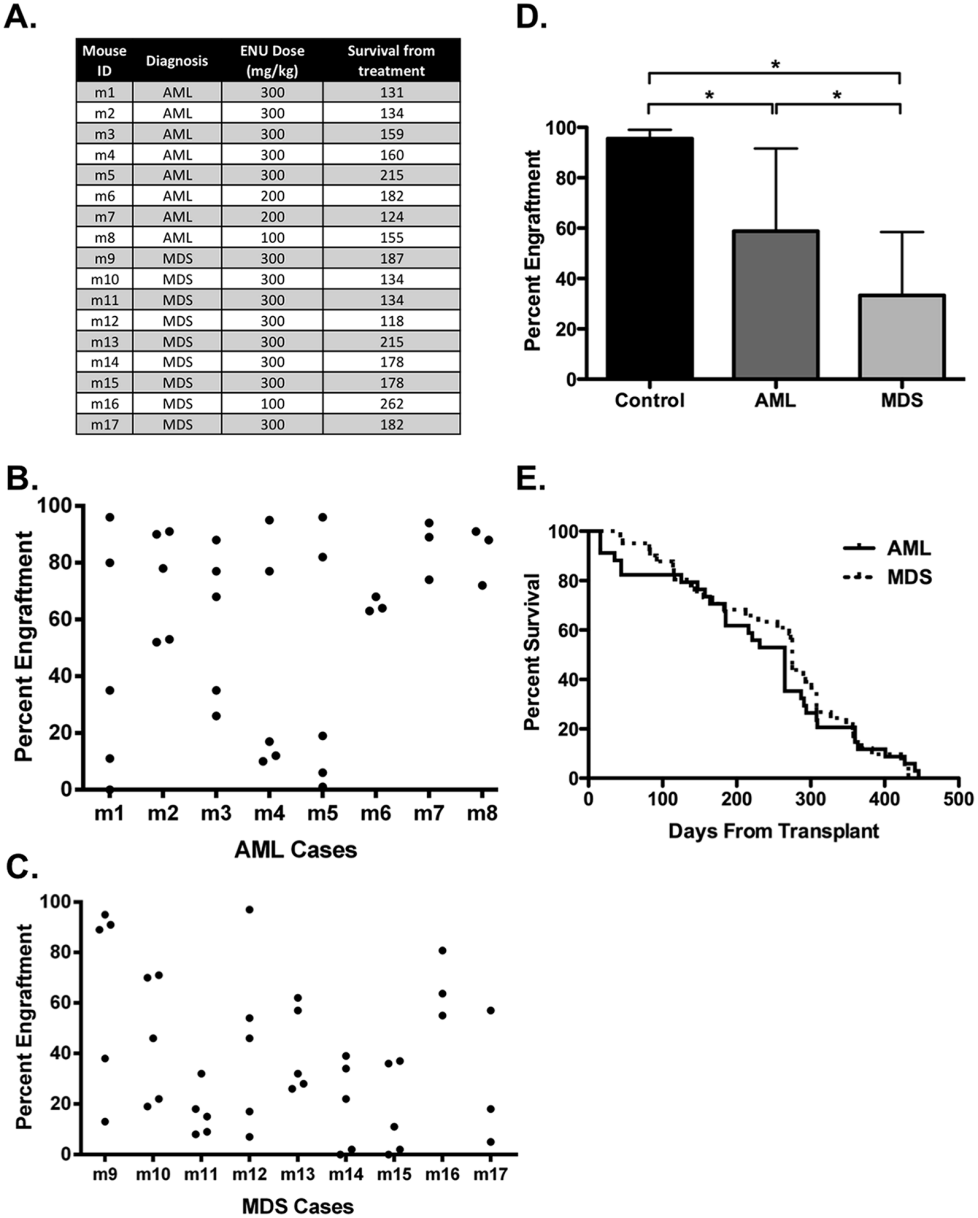
AML and MDS cases engraft NSG mice but do not transplant rapidly lethal disease. Single-cell suspensions of bone marrow cells from diseased mice were prepared at sacrifice and transplanted either as bulk or CD3-depleted samples into conditioned NSG mice (n = 3 or 5 transplants per primary mouse). Peripheral blood engraftment was monitored by flow cytometry using congenic CD45.1 (NSG) and CD45.2 (DBA/SWR) markers. **(a)** A summary of ENU-treated mice used in transplant experiments, including mouse ID, diagnosis, ENU dose received, and survival from the time of ENU-treatment is shown. **(b, c)** Scatter plots of peripheral blood engraftment 60 days after transplant, expressed as the percentage of CD45.2 positive cells out of total CD45 positive cells, is shown for eight AML **(b)** and nine MDS **(c)** cases. **(d)** Mean PB engraftment with standard deviations of all AML (n = 34) and MDS (n = 47) transplants at 60 days is shown compared to transplants from two untreated age-matched control mice. AML cases engrafted at a significantly higher percentage than MDS cases, and both AML and MDS mean engraftment levels were significantly lower than control (* p<0.05 by unpaired t test). **(e)** Kaplan-Meier survival analysis of transplanted mice demonstrates a long median overall survival for both AML (n = 34, 265 days) and MDS (n = 47, 275 days) transplants.

According to the MMHCC, one of the defining criteria for nonlymphoid leukemia is transplantability to normal or sublethally irradiated recipients and lethality in transplant recipients with a median time of eight weeks or less from transplantation to death [47]. In addition, transplantation assays allow for identification and study of LSC. Therefore, we next tested whether our ENU-induced mouse AML and MDS models could engraft sublethally irradiated adult NSG mice and whether such engraftment was rapidly fatal to the recipient mice. Seventeen primary disease models (eight AML and nine MDS) were selected for this study and primary animal data, including ENU dose and survival from treatment, are summarized in Fig 5A and Table B in S1 File. Two primary MDS mice were not used in these experiments because of inadequate sample. We first screened for ability to engraft secondary recipient mice by transplanting 1–5 x 10^6^ bulk BM cells from each primary mouse into 3–5 sublethally irradiated adult NSG mice. In some cases, we first FACS purified CD3-depleted BM from primary mice prior to transplantation. Mice were monitored for signs of disease, and PB engraftment was assayed every four weeks by flow cytometry using congenic CD45.1 (NSG) and CD45.2 (DBA/SWR) markers. By day 60 after transplant, peripheral blood engraftment was detected in at least two mice from each primary AML (Fig 5B) and MDS (Fig 5C) transplant. Levels of engraftment were generally stable over the lifespan of the recipient mouse. PB engraftment at day 60 after transplant was significantly higher from primary AML mice compared to primary MDS mice (Fig 5D, p<0.05). Both AML and MDS mice had significantly decreased engraftment compared to control transplants using age-matched normal BM (Fig 5D, p<0.05). The median overall survival for NSG mice transplanted with AML and MDS was 265 and 275 days respectively, which was not significantly different (Fig 5E). Of the 34 secondary mice transplanted with AML and 47 transplanted with MDS, only six AML and two MDS secondary mice died from transplanted disease within eight weeks of transplant. Upon sacrifice, these mice exhibited clinical and morphologic features of acute leukemia, although the disease immunophenotype in the secondary animal differed from the primary animal in some cases (Table B in S1 File). Similarly, among the secondary mice that died after eight weeks from transplant, many died with clinical and morphologic features of acute leukemia. However, these mice demonstrated a wide spectrum of disease immunophenotypes that were often distinct from the primary disease (Table B in S1 File). A summary of the entire cohort of secondary mice used in these experiments is shown in Table B in S1 File. Overall, our ENU-induced mouse models of AML and MDS were capable of engraftment in NSG mice, but were not rapidly lethal to the transplant recipient.

To determine which BM cell subpopulation was responsible for establishing secondary transplants from primary mice with ENU-induced AML and MDS, we next FACS purified four BM cell subpopulations including the HSC-containing KLS positive and hematopoietic progenitor cell-containing KLS negative cells as well as the Lineage positive and Lineage negative/c-Kit negative (“c-Kit negative”) cell subpopulations (Figure A in S1 File). These BM subpopulations were isolated from three mice with AML (m6, m7, and m8 in Fig 5A) and two mice with MDS (m11 and m17 in Fig 5A), and each subpopulation was transplanted into two sublethally irradiated adult NSG mice. Equal numbers of cells were transplanted from each cell subpopulation for each primary mouse (range 434–5736 cells per transplant). Mice were monitored for signs of disease and PB engraftment was assayed every four weeks by flow cytometry using congenic CD45.1 (NSG) and CD45.2 (DBA/SWR) markers. At day 60 after transplant, sustained engraftment was only seen in transplants from the HSC-containing KLS positive BM subpopulation (Figure A in S1 File). The two KLS positive transplants with minimal (1–3%) engraftment were from MDS m11, which had the fewest transplanted cells (434) per secondary recipient. Stable engraftment was seen in both bulk and KLS positive transplants when recipient mice were followed out to 180 days post-transplant (Figure A in S1 File). For both bulk and KLS positive transplants, the percentages of c-Kit positive and myeloid marker positive cells trended down over time while the level of lymphoid engraftment, in particular T cells, increased (Figure A in S1 File). None of the KLS positive transplant recipient mice died from rapidly fatal disease or exhibited clear clinical findings consistent with AML, MDS, or graft-versus-host disease apart from rare dysplastic cells of unclear significance. Overall, KLS positive and bulk bone marrow cells from ENU-induced AML and MDS mouse models were capable of establishing secondary transplants, but did not lead to rapidly fatal disease or recapitulation of the primary disease phenotype.

### Primary human t-AML samples can form successful xenografts characterized by lethality to recipient mice and variable LSC immunophenotype

**Fig 6:**
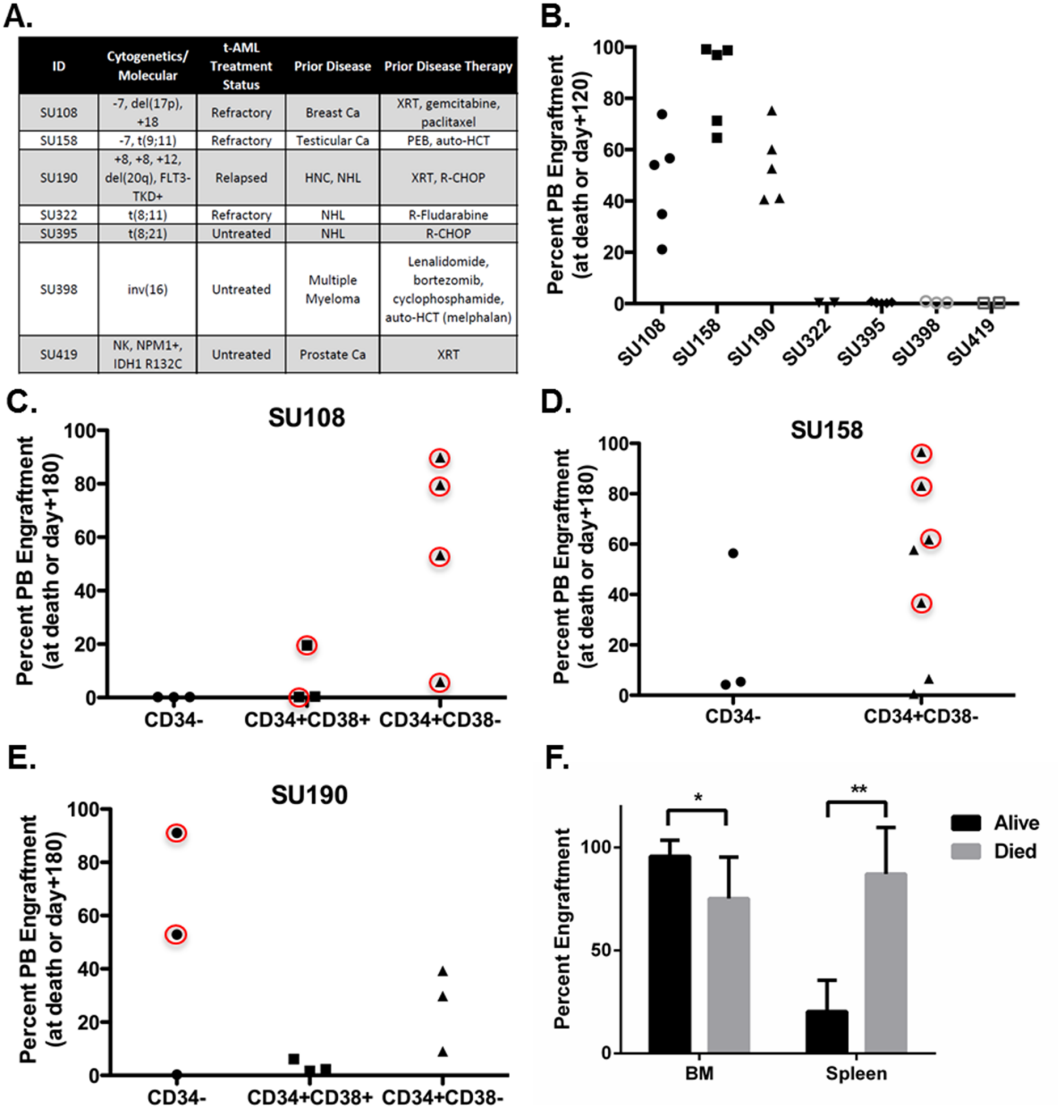
Human t-AML samples can form successful xenografts in NSG mice, but do not demonstrate a uniform LSC immunophenotype. CD3-depleted human AML cells were transplanted into conditioned NSG mice either as bulk or FACS purified CD34-, CD34+/CD38+, and CD34+/CD38- cell subpopulations. Peripheral blood engraftment was monitored by flow cytometry using CD45.1 (NSG) and hCD45 (human) markers. **(a)** A summary of patient disease-related information including cytogenetics and molecular alterations, disease status, prior disease, and prior therapy is shown. **(b-e)** Scatter plots of percentage PB engraftment, expressed as the percentage of hCD45 positive cells out of total CD45 positive cells, at day 120 or death **(b)** or at day 180 or death **(c-e)**, are shown. **(b)** CD3-depleted bulk AML samples from the seven t-AML cases were transplanted into conditioned NSG mice (n = 2–5 per primary t-AML sample). Three samples (SU108, SU158, and SU190) established hCD45+hCD33+ AML xenografts with moderate to high engraftment that were all fatal to the recipient mice by 127 days after transplantation. CD34-, CD34+/CD38+, and CD34+/CD38- cell subpopulations from engrafting samples (SU108, SU158, and SU190) were FACS purified and transplanted into conditioned NSG mice (n = 3–7 per cell subpopulation from each primary t-AML sample). **(c)** CD34+/CD38+ and CD34+/CD38-, but not CD34-, cells from SU108 established AML xenografts. Xenografts from both cell subpopulations were fatal to the recipient mice (circled). **(d)** Both CD34- and CD34+CD38- cells from SU158 established AML xenografts that were fatal to the recipient in three of seven mice from the CD34+CD38- cell subpopulation (circled). **(e)** All three cell subpopulations from SU190 were capable of forming AML xenografts, although only CD34- transplanted fatal disease (circled). **(f)** Mice with nonlethal engraftment had significantly higher BM engraftment and lower spleen engraftment than mice that died from t-AML xenografts (* p = 0.00396, ** p = 7.235 x 10^−8^ by Student’s t-test).

Since there are few previously described human t-AML models, we next attempted to model human t-AML in patient-derived xenograft NSG mouse models to complement our ENU-induced mouse AML/MDS models. We identified seven cases of WHO-defined t-AML from our tissue bank. Clinical information is summarized in Fig 6A. Each patient received prior radiation and/or chemotherapy associated with development of t-AML. We first screened for ability to form xenografts by transplanting 1–5 x 10^6^ CD3-depleted bulk AML cells into sublethally irradiated adult NSG mice. Mice were monitored for signs of disease and PB engraftment was assayed every four weeks by flow cytometry using CD45.1 (NSG) and hCD45 markers. Composition of engrafted hCD45-positive cells was determined by staining with CD33 (myeloid, positive in all primary t-AML samples used here), CD19 (B cell), and CD3 (T cell). PB engraftment at the time of NSG mouse death from t-AML or day 120 from transplant is shown in Fig 6B. Three out of the seven primary t-AML samples tested were able to establish hCD45/hCD33-positive xenografts in 5/5 NSG mice (SU108, SU158, and SU190). Interestingly, the three primary t-AML samples that formed xenografts all displayed high-risk cytogenetics and were collected from patients with t-AML that was relapsed after or refractory to anti-AML chemotherapy (Fig 6A). Consistent with these adverse clinical features, all three primary samples led to aggressive disease in the xenograft model. All mice carrying xenografts with SU108, SU158, and SU190 died within 67, 127, and 31 days after transplant respectively, and these xenografted mice were characterized by severe cytopenias with circulating blasts, splenomegaly, high engraftment in both the bone marrow and spleen, and infiltration of the liver (Figure B in S1 File and Table C in S1 File).

Since the immunophenotype of LSC in t-AML is not well-described, we next FACS purified CD34-, CD34+/CD38+, and CD34+/CD38- subpopulations from SU108, SU158, and SU190 and transplanted equal numbers of these cell subpopulations into sublethally irradiated adult NSG mice (3–7 mice per subpopulation per primary AML sample) to identify subpopulations capable of initiating AML xenografts. Mice were monitored as above until the time of death or 180 days from transplant. CD34+CD38+ and CD34+CD38- cells, but not CD34- cells, from SU108 were able to establish xenografts (Fig 6C). One mouse from each engrafting population died unexpectedly and engraftment analysis at death could not be performed. Both mice had little peripheral blood engraftment at the previous day 60 check. The remaining two CD34+CD38+ and all three of the CD34+CD38- mice exhibited engraftment that led to death in the recipient NSG mice by day 141 after transplant. These mice were characterized by severe cytopenias and high engraftment with hCD45/hCD33+ cells in the BM and spleen. SU158 did not have a distinct CD34+CD38+ cell subpopulation so only CD34- and CD34+CD38- cells were isolated by FACS. All mice transplanted with CD34- or CD34+CD38- cells from SU158 established xenografts (Fig 6D). Lethal disease was only demonstrated in mice transplanted with CD34+CD38- cells (circled in Fig 6D). Unlike SU108 and SU158, only CD34- cells from SU190 led to fatal disease in recipient mice although all three subpopulations were capable of establishing xenografts (Fig 6E). In all three t-AML xenograft models, mice that survived to 180 days from transplant were also sacrificed and their BM and spleens were evaluated for engraftment. Mice that had no PB engraftment also did not have engraftment in the BM or spleen. Interestingly, mice that died from t-AML xenografts (circled in Fig 6C–6E) had high levels of hCD45/hCD33+ engraftment in both the BM and spleen; whereas, engrafted mice that did not die from t-AML xenografts had high levels of engraftment in the BM but low levels of engraftment in the spleen (Fig 6F). BM engraftment was significantly lower in mice with fatal t-AML xenografts suggesting that effacement of the normal mouse spleen by the xenograft is critical for determining the survival of the recipient mouse (Fig 6F). Mice that succumbed to t-AML xenografts were also characterized by splenomegaly, weight loss, anemia, and thrombocytopenia. Conversely, mice with nonlethal engraftment had only mild anemia. A summary of the clinical and engraftment data from NSG mice transplanted with bulk or FACS purified cell subpopulations from SU108, SU158 and SU190 is shown in Table C in S1 File. Overall, we were able to establish three distinct patient-derived xenograft models of t-AML each with differences in immunophenotypic subpopulations capable of establishing xenografts with varying BM engraftment, spleen engraftment, and survival.

## Discussion

t-AML/MDS is a distinct subgroup of AML that develops years after exposure to chemotherapy and ionizing radiation. These diseases are characterized clinically by recurrent chromosomal alterations and gene mutations, and carry a dismal prognosis with current treatments. Overall, less is known about t-AML/MDS pathogenesis compared to *de novo* disease, and relatively few t-AML/MDS mouse models have been described. Here, we describe multiple mouse models of t-AML/MDS with features consistent with clinical disease. We first developed models of alkylator-induced t-AML/MDS by treating wild type adult DBA/2J and SWR/J mice with ENU and established a diagnosis of AML or MDS in nineteen mice using the rigorous diagnostic criteria proposed by the MMHCC. We demonstrated that these models have clinical and pathologic characteristics consistent with human t-AML/MDS and are therefore relevant models to study human disease. We also established xenograft models derived from three primary human t-AML samples. These models were characterized by lethality to recipient mice and LSC immunophenotype that varied among the primary t-AML cases.

Our mouse alkylator-induced AML and MDS models closely modeled many clinical features of human t-AML/MDS. Mice developing AML or MDS became rapidly moribund with leukocytosis, anemia, and thrombocytopenia in AML cases and macrocytic anemia and thrombocytopenia in MDS cases. The mouse diseases characterized here also had morphologic features consistent with human disease, and the bone marrow was hypercellular with expanded myeloid cell populations as in human AML and MDS. The finding of decreased immunophenotypic HSC in MDS mice without changes in other HSPC compartments differs from prior reports showing expansion of HSC and CMP [48] or GMP [14] in higher-risk MDS and could represent a novel finding in t-AML/MDS or a species-specific phenomenon. Interestingly, adjusting the dose of ENU favored the development of myeloid diseases in mice that survived acute toxicity at higher ENU doses while lower ENU doses favored development of lymphoid diseases, suggesting ENU can be used to prepare custom models of hematopoietic diseases in DBA/2J and SWR/J mice. The median survival of 162 days from treatment for ENU-induced AML and MDS further establishes this model as a practical research tool. Limitations of our model included inability to transplant fatal disease to recipient mice and difficulty identifying clonal markers. We demonstrated that all AML and MDS cases were capable of transplanting immune deficient mice. Only the KLS positive HSPC subpopulation contained stem cell activity, but these cells could not fully recapitulate the aggressive nature of the primary disease, particularly in the AML cases. We observed the same finding when we transplanted primary DBA/2J AML or MDS cells into irradiated DBA/2J mice (data not shown). There are multiple interpretations of the inability of our ENU models to transplant aggressive disease and the immunophenotypic variability in recipient mice: 1) Given the oligoclonality seen in AML and MDS, it is possible that our ENU-induced AML/MDS models are also oligoclonal and clones were randomly selected for in recipient mice; 2) The differences seen in secondary mice were the result of clonal evolution from the primary transplanted clone; and 3) The primary disease did not engraft and an abnormal non-AML/MDS clone was transplanted and evolved to a frank disease over time in each recipient mouse [49, 50]. Our findings differ from a recently described ENU-induced AML model in SWR/J mice [45] and could reflect differences in experimental design or technique. The difficulty in establishing secondary transplants from primary AML and MDS is well described and the lack of lethality to transplant recipients with our MDS model is consistent with these other studies [13, 14, 17, 51, 52]. Although clonal markers are not required for diagnosis of mouse AML or MDS [47], we attempted to identify a clonal marker in one of our disease models by performing exome sequencing (data not shown). We observed a much higher than predicted rate of ENU mutagenesis with 5629 cancer-specific nucleotide variants compared to control [53, 54]. Validation of relevant clonal markers from such a high number of potential mutations was beyond the scope of this study.

Since the AML LSC model has been most well-described in *de novo* AML, we attempted to establish xenografts with a panel of seven primary human t-AML samples. Three primary samples led to engraftment of aggressive and rapidly lethal disease in NSG mice. Interestingly, these three patients had adverse-risk complex or monosomal karyotypes and refractory disease consistent with prior studies showing that the likelihood of establishing AML xenografts correlates with karyotype and treatment response [51]. When we examined subpopulations from these three primary t-AML samples, we found no unifying immunophenotype capable of engrafting t-AML into NSG mice and each of CD34-, CD34+CD38+, and CD34+CD38- were capable of establishing xenografts from one or more of the primary samples. Thus, this small cohort suggests there may be no canonical LSC population in t-AML. Intriguingly, some xenografted mice developed rapidly fatal disease while others had sustained nonlethal engraftment. Morphologic and immunophenotypic evaluation of the BM and spleen revealed that all engrafted mice had significant levels of BM engraftment, but effacement of the spleen with human AML characterized mice that succumbed to AML. Therefore, within each primary human t-AML sample, multiple cell populations with LSC activity existed that had differential BM and splenic engrafting activity and resultant lethality to the recipient mice. The existence of multiple hierarchical LSC populations in individual AML samples has been described [31], and it is possible our xenograft models show a hierarchical relationship between LSC population and lethal phenotype that does not conform to classic immunophenotypic hierarchies. It also suggests that LSC-defining experiments can miss cell subpopulations with LSC activity depending on how engraftment in the recipient mouse is determined. Although it is possible this finding is an experimental artifact, it does raise questions about whether the currently accepted AML LSC model addresses potential differences in phenotype of transplanted cell subpopulations and whether these differences are biologically or clinically relevant. Overall, because of the small sample size, these results are primarily hypothesis-generating and additional analyses with larger numbers of primary human t-AML samples are required to confirm our findings.

In summary, we report several new mouse models of t-AML/MDS that are consistent with clinical disease and that could potentially be used to further define disease susceptibility and pathogenesis and test novel therapeutics. For example, future studies could take advantage of the predictable timing of MDS development to evaluate therapeutic and transplant approaches or tailor ENU dosage to study specific hematopoietic diseases. Further analysis of human t-AML LSC should also confirm the heterogeneous immunophenotypic and mouse phenotypic characteristics of human t-AML LSC seen in our models.

## Supporting Information

S1 File Jonas et al. Supporting Information. Figure A. KLS positive cells from AML and MDS cases stably engraft NSG mice with lymphoid-biased hematopoiesis over time. Figure B. Human t-AML samples form PDX models in NSG mice. Table A. Strain, Gender, ENU Dose, Diagnosis, Survival, CBC, Necropsy and Immunophenotype for all primary mice. Table B. Characteristics of secondary transplant mice described in Fig 5. Table C. Characteristics of mice transplanted with human t-AML samples (SU108, SU158 and SU190).
(PDF)
