# Supplementary material for: Alkylator-Induced and Patient-Derived Xenograft Mouse Models of Therapy-Related Myeloid Neoplasms Model Clinical Disease and Suggest the Presence of Multiple Cell Subpopulations with Leukemia Stem Cell Activity
Source: PLoS One. 2016 Jul 18;11(7):e0159189. doi: 10.1371/journal.pone.0159189 (PMC4948781; doi:10.1371/journal.pone.0159189)
Supplement: S1 File — Figure A. KLS positive cells from AML and MDS cases stably engraft NSG mice with lymphoid-biased hematopoiesis over time. Figure B. Human t-AML samples form PDX models in NSG mice. Table A. Strain, Gender, ENU Dose, Diagnosis, Survival, CBC, Necropsy and Immunophenotype for all primary mice. Table B. Characteristics of secondary transplant mice described in Fig 5. Table C. Characteristics of mice transplanted with human t-AML samples (SU108, SU158 and SU190). (PDF) [file pone.0159189.s001.pdf]

S1 File. Jonas et al. Supporting Information.

Figure A. KLS positive cells from AML and MDS cases stably engraft NSG mice with lymphoid-biased hematopoiesis over time.

Figure B. Human t-AML samples form PDX models in NSG mice.

Table A. Strain, Gender, ENU Dose, Diagnosis, Survival, CBC, Necropsy and Immunophenotype for all primary mice.

Table B. Characteristics of secondary transplant mice described in Fig 5.

Table C. Characteristics of mice transplanted with human t-AML samples (SU108, SU158 and SU190).

FIGURE A

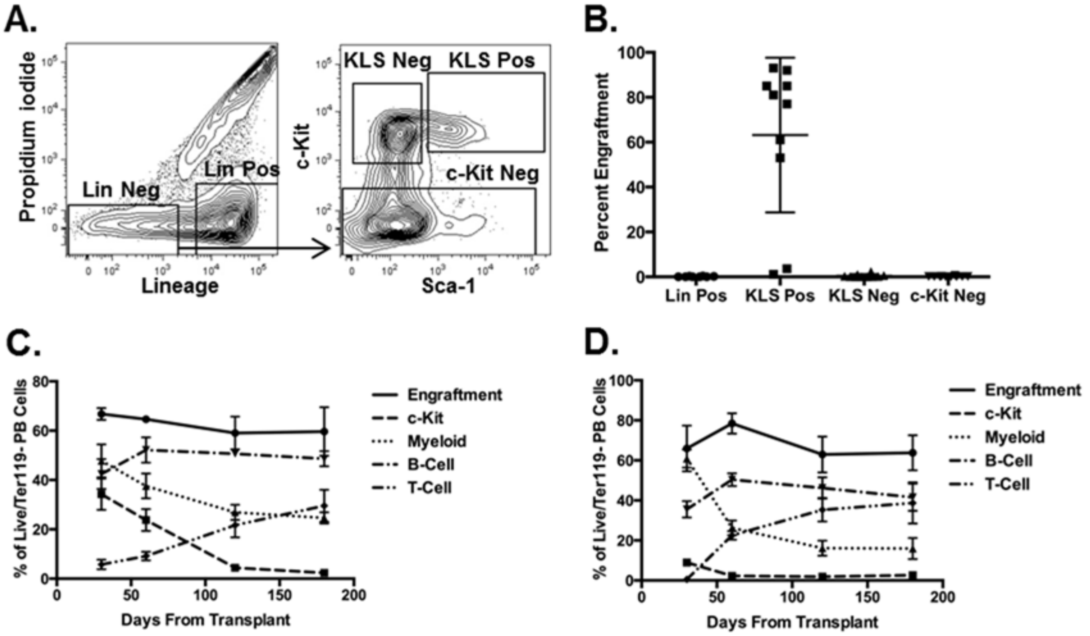

Figure A. KLS positive cells from AML and MDS cases stably engraft NSG mice with lymphoid-biased hematopoiesis over time. Lineage positive, c-Kit negative, KLS negative, and KLS positive bone marrow HSPC subpopulations were isolated by FACS purification and transplanted into conditioned NSG mice. **(a)** The gating strategy for HSPC transplants is shown. KLS positive, KLS negative, and c-Kit negative subpopulations were isolated from the Lineage negative gate. **(b)** Scatter plots with means and standard deviations of PB engraftment at 60 days post-transplant, expressed as the percentage of CD45.2 positive cells out of total CD45 positive cells, is shown for transplants of Lineage positive (n = 8), KLS positive (n = 10), KLS negative (n = 10), and c-Kit negative (n = 10) BM cell subpopulations. Sustained peripheral blood engraftment was only seen in transplants containing KLS positive cells. **(c, d)** Means and standard deviations of percentages of PB engraftment and of live/Ter119 negative cells expressing myeloid and lymphoid markers at 30, 60, 120, and 180 days post-transplantation are shown for bulk BM **(c)** and KLS positive **(d)** transplants. Both bulk and KLS positive transplants show stable engraftment up to 180 days after transplant, with relative numbers of c-Kit positive and myeloid cells decreasing and lymphoid cells increasing over time.

FIGURE B

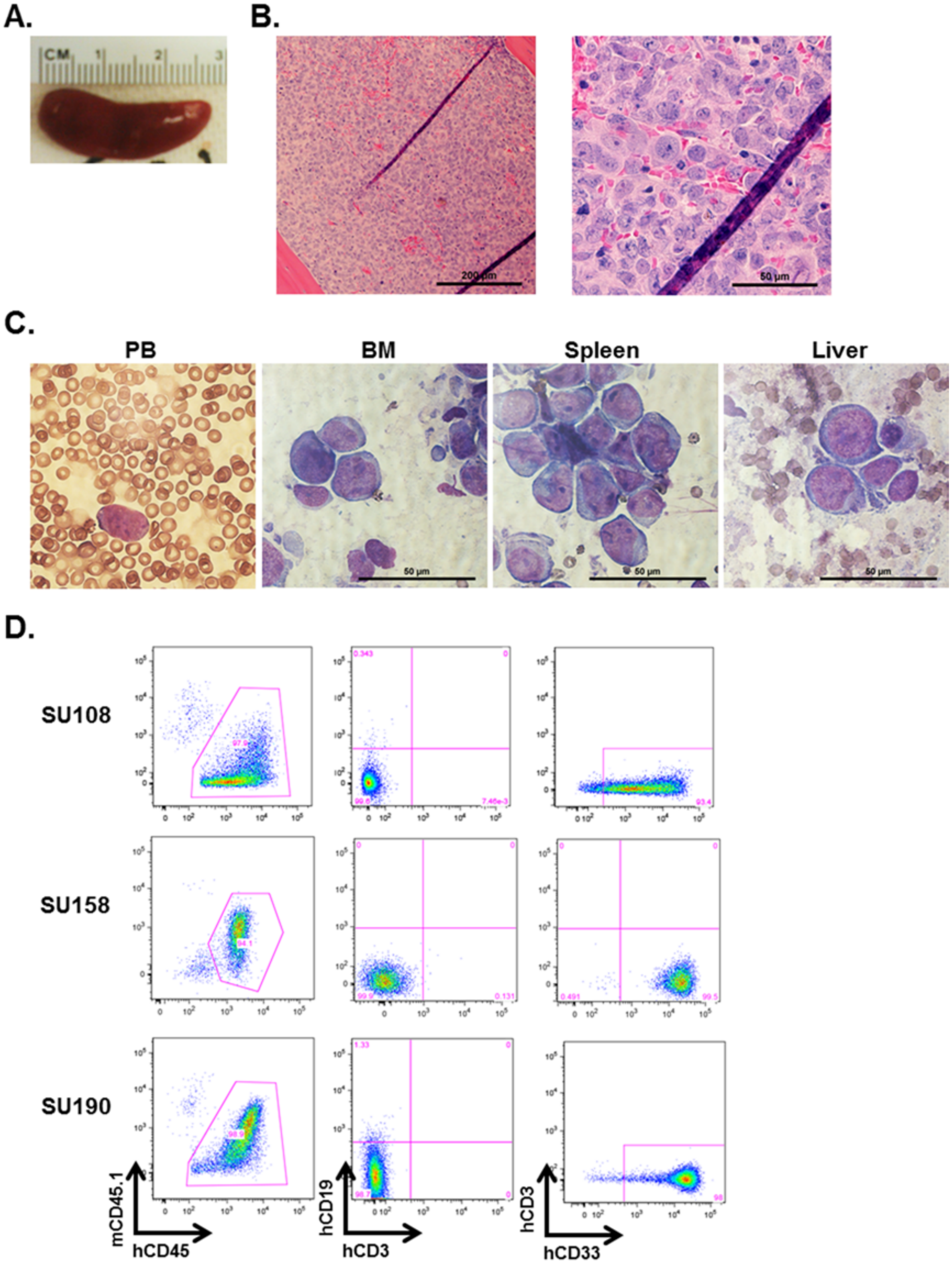

A.
B.
C.
D.
CM
200 µm
50 µm
PB
BM
Spleen
Liver
SU108
SU158
SU190
mCD45.1
hCD45
hCD19
hCD3
hCD3
hCD33

Figure B. Human t-AML samples form PDX models in NSG mice. (a) A representative example of splenomegaly from an NSG mouse engrafted with SU108 is shown. The spleen is markedly enlarged compared to normal NSG mice consistent with engraftment of human t-AML. **(b)** A representative example of bone marrow infiltration by AML from an NSG mouse engrafted with SU108 is shown. Images are from an H&E stained decalcified FFPE tibia specimen. The left panel is at 100x magnification and the right panel is at 400x magnification. The normal NSG marrow is effaced with large immature mononuclear cells consistent with engraftment of human t-AML. **(c)** Representative smears (630x) from Harleco Hemacolor Staining System-stained PB, BM, spleen and liver of NSG mice engrafted with SU108 are shown. Circulating large immature mononuclear cells are present in the PB and the BM, spleen and liver are infiltrated by large immature mononuclear cells consistent with engraftment of human t-AML. **(d)** Representative flow cytometry dot plots showing engraftment of NSG mouse bone marrow with SU108, SU158 and SU190 are shown. In each example, there is high engraftment with hCD45+/hCD33+ cells, consistent with engraftment of human t-AML.

Table A. Strain, Gender, ENU Dose, Diagnosis, Survival, CBC, Necropsy and Immunophenotype for all primary mice.

Mouse ID	Fig5 Mouse ID	Strain	Sex	ENU Dose (mg/kg)	Diagnosis	Survival Post ENU (d)	Weight (g)	WBC (5-12)	Hgb (13-16)	Plts (300-1000)	MCV (42.3-55.9)	Necropsy Findings	Main BM Immunophenotype
m1-1		DBA/2J	M	300	T-ALL	178	21.6	21.1	1.4	24	51.8	Splenomegaly (2.1cm)	c-Kit+/CD4+/CD8+
m1-2	m1	DBA/2J	M	300	AML	131	32.3	13.0	12.4	432	47.6	Found Dead, Splenomegaly (1.5cm)	c-Kit+/Gr-1+/Mac-1+
m1-3	m9	DBA/2J	M	300	MDS	187	25.9	8.3	4.9	189	47.0	Splenomegaly (2.7cm)	c-Kit+/Gr-1+/Mac-1+
m1-4		DBA/2J	M	300	B-ALL	134	28.0	19.9	8.9	169	50.3	Splenomegaly (2.5cm)	c-Kit+/CD19+/CD20+
m1-5	m2	DBA/2J	M	300	AML	134	29.4	23.9	7.2	209	45.1	Splenomegaly (2.4cm)	c-Kit+/Gr-1+/Mac-1+
m2-1	m10	DBA/2J	F	300	MDS	134	26.7	35.5	6.5	337	42.7	Splenomegaly (2.4cm)	Gr-1+/Mac-1+
m2-2	m11	DBA/2J	F	300	MDS	134	20.6	5.2	4.6	160	51.0	Splenomegaly (2cm)	Gr-1+/Mac-1+
m2-3	m3	DBA/2J	F	300	AML	159	22.2	22.1	7.6	170	48.8	Found Dead, Splenomegaly (2cm)	c-Kit+/Gr-1+/Mac-1+
m2-4	m4	DBA/2J	F	300	AML	160	18.8	28.3	3.7	183	45.3	Splenomegaly (2.6cm)	Gr-1+/Mac-1+
m2-5		DBA/2J	F	300	MPN	189	21.6	15.7	4.7	335	43.7	Splenomegaly (2.7cm)	Gr-1+/Mac-1+
m3-1	m12	SWR/J	M	300	MDS	118	25.0	15.2	9.6	407	48.4	Splenomegaly (1.5cm), Bladder stones	c-Kit+/Gr-1+/Mac-1+
m3-2		SWR/J	M	300	ENU Toxicity	21	ND	ND	ND	ND	ND	Found Dead	ND
m3-3		SWR/J	M	300	T-ALL	168	25.0	41.2	8.3	55	49.7	Splenomegaly (3cm), Mediastinal Mass	CD4+/CD8+
m3-4	m5	SWR/J	M	300	AML	215	22.5	20.5	3.5	172	76.8	Splenomegaly (2.7cm), Rectal Mass	c-Kit+/Gr-1+/Mac-1+
m3-5	m13	SWR/J	M	300	MDS	215	26.2	12.2	5.2	177	65.0	Splenomegaly (2.5cm)	c-Kit+/Gr-1+/Mac-1+
m4-1		SWR/J	F	300	ENU Toxicity	24	ND	ND	ND	ND	ND	Found Dead	ND
m4-2	m14	SWR/J	F	300	MDS	178	23.9	5.4	10.3	219	59.3	Found Dead, Splenomegaly (2.5cm), Abdominal Tumor	c-Kit+/Gr-1+
m4-3		SWR/J	F	300	ENU Toxicity	21	ND	ND	ND	ND	ND	Found Dead	ND
m4-4	m15	SWR/J	F	300	MDS	178	19.6	14.2	5.1	401	61.2	Splenomegaly (2cm)	Gr-1+/Mac-1+
m4-5		SWR/J	F	300	Unknown	220	24.6	15.2	7.2	509	67.9	Found Dead, Lung Masses	ND
m7-1		DBA/2J	M	100	MDS	328	39.1	14.6	12.0	102	44.8	Splenomegaly (1.7cm), Eye Tumor	Gr-1+/Mac-1+
m7-2		DBA/2J	M	100	MDS	340	35.7	20.1	11.3	402	45.7	Splenomegaly (2cm), Abdominal Tumor	Gr-1+/Mac-1+
m7-3	m16	DBA/2J	M	100	MDS	262	33.7	9.1	9.0	117	51.0	Splenomegaly (1.8cm)	Gr-1+/Mac-1+
m7-4	m8	DBA/2J	M	100	AML	155	28.2	11.8	14.3	186	45.2	Splenomegaly (1.5cm), Mediastinal Mass	c-Kit+/Gr-1+/Mac-1+
m7-5		DBA/2J	M	100	T-ALL	113	27.9	46.7	2.9	222	45.8	Found Dead, Splenomegaly (1.5cm), Mediastinal Mass	c-Kit+
m8-1		DBA/2J	M	300	ENU Toxicity	11	ND	ND	ND	ND	ND	Unremarkable	ND
m8-2		DBA/2J	M	300	ENU Toxicity	12	ND	ND	ND	ND	ND	Unremarkable	ND
m8-3		DBA/2J	M	300	ENU Toxicity	13	ND	ND	ND	ND	ND	Unremarkable	ND
m8-4		DBA/2J	M	300	ENU Toxicity	13	ND	ND	ND	ND	ND	Unremarkable	ND
m8-5		DBA/2J	M	300	ENU Toxicity	13	ND	ND	ND	ND	ND	Unremarkable	ND
m9-1	m6	DBA/2J	M	200	AML	182	28.6	11.4	8.3	428	45.7	Splenomegaly (2.6cm)	c-Kit+/Gr-1+/Mac-1+
m9-2		DBA/2J	M	200	T-ALL	145	26.0	12.1	6.9	54	54.8	Splenomegaly (4cm), Adenopathy	Gr-1+/TCRb+/CD3+/CD4+/CD8+
m9-3		DBA/2J	M	200	T-ALL	162	24.0	16.7	4.6	86	52.2	Splenomegaly (3cm), Adenopathy	Gr-1+/TCRb+/CD3+/CD4+/CD8+
m9-4	m7	DBA/2J	M	200	AML	124	28.0	13.4	12.0	338	45.7	Splenomegaly (2.1cm)	Gr-1+/Mac-1+
m9-5		DBA/2J	M	200	Mast Cell Sarcoma	272	25.4	25.9	8.6	373	56.5	Splenomegaly (1.8cm), Kidney enlargement	c-Kit+/Mac-1+
m10-1		DBA/2J	F	100	T-ALL	155	24.5	54.3	5.7	255	47.2	Found Dead, Splenomegaly (1.6cm), Mediastinal Mass	c-Kit+
m10-2		DBA/2J	F	100	T-ALL	151	22.5	31.5	9.5	97	41.5	Splenomegaly (3.5cm), Hepatomegaly, Adenopathy	c-Kit+/CD4+/CD8+
m10-3		DBA/2J	F	100	T-ALL	151	26.1	31.5	9.5	139	44.8	Splenomegaly (3.5cm), Hepatomegaly, AdenopathyMediastinal Mass	c-Kit+/CD8+
m10-4		DBA/2J	F	100	T-ALL	227	25.6	3.9	6.5	43	43.5	Splenomegaly (3.6cm), Hepatomegaly, Adenopathy	c-Kit+
m10-5		DBA/2J	F	100	ENU Toxicity	30	ND	ND	ND	ND	ND	Found Dead	ND
m11-1		DBA/2J	F	300	ENU Toxicity	18	ND	ND	ND	ND	ND	Unremarkable	ND
m11-2		DBA/2J	F	300	ENU Toxicity	13	ND	ND	ND	ND	ND	Unremarkable	ND
m11-3	m17	DBA/2J	F	300	MDS	182	15.3	10.4	11.1	527	48.2	Splenomegaly (1.7cm)	c-Kit+/Gr-1+/Mac-1+
m11-4		DBA/2J	F	300	Ovarian Carcinoma	206	17.6	10.1	4.1	107	56.4	Splenomegaly (2.5cm), Ovarian Tumor	Gr-1+/Mac-1+
m11-5		DBA/2J	F	300	ENU Toxicity	8	ND	ND	ND	ND	ND	Found Dead	ND
m12-1		DBA/2J	F	200	T-ALL	182	22.7	35.4	12.2	161	48.1	Splenomegaly (3.5cm), Adenopathy, Mediastinal Mass	c-Kit+/CD4+/CD8+
m12-2		DBA/2J	F	200	T-ALL	156	22.3	33.3	7.8	59	57.2	Splenomegaly (3.3cm), Hepatomegaly, Mediastinal Mass	TCRb+/CD3+/CD4+/CD8+
m12-3		DBA/2J	F	200	Unknown	249	22.2	7.6	15.1	300	45.0	Found Dead, Splenomegaly (2cm)	ND
m12-4		DBA/2J	F	200	Unknown	144	28.7	10.5	14.4	258	46.5	Found Dead, Splenomegaly (2cm), Adenopathy, Mediastinal Mass	ND
m12-5		DBA/2J	F	200	T-ALL	156	18.6	5.2	12.9	134	47.4	Splenomegaly (2.4cm), Adenopathy, Mediastinal Mass	c-Kit+/CD4+/CD8+

ND = No data

Normal ranges indicated for WBC, Hgb, Plt and MCV

Table B. Characteristics of secondary transplant mice described in Fig 5.

Secondary Mouse ID	Primary Mouse ID (Fig 5)	Strain	ENU Dose (mg/kg)	Diagnosis	Survival	Primary Mouse Immunophenotype	Donor Cell Type, Dose	Mouse Fate	Survival After Transplant	Weight	WBC	Hgb	Plt	Necropsy	PB Engraftment	Immunophenotype
m1-1	m1	DBA/2J	300	AML	131	c-Kit+/Gr-1+/Mac-1+	Bulk, 3e6	SAC	366	27.4	1.8	8.6	314	Splenomegaly, Mediastinal Mass	56.0	Lin-
m1-2	m1	DBA/2J	300	AML	131	c-Kit+/Gr-1+/Mac-1+	Bulk, 3e6	SAC	44	18.6	2.2	8.4	93	Splenomegaly	96.1	CD3 lo
m1-3	m1	DBA/2J	300	AML	131	c-Kit+/Gr-1+/Mac-1+	Bulk, 3e6	SAC	44	18.6	1.6	9.7	156	Splenomegaly	80.3	CD3 lo
m1-4	m1	DBA/2J	300	AML	131	c-Kit+/Gr-1+/Mac-1+	T-cell deplete bulk, 5e6	SAC	164	19.0	48.7	10.6	168	Splenomegaly, Mediastinal Mass	95.1	CD3 lo
m1-5	m1	DBA/2J	300	AML	131	c-Kit+/Gr-1+/Mac-1+	T-cell deplete bulk, 5e6	EOE	308	26.6	1.6	13.1	333	Unremarkable	0.5	Lin-
m2-1	m2	DBA/2J	300	AML	134	c-Kit+/Gr-1+/Mac-1+	Bulk, 5e6	SAC	265	23.9	18.8	12.5	258	Splenomegaly, Flank Tumor	97.6	c-Kit+/B220+
m2-2	m2	DBA/2J	300	AML	134	c-Kit+/Gr-1+/Mac-1+	Bulk, 5e6	FD	401	27.5	4.0	10.2	330	ND	93.8	Gr-1+/Mac-1+
m2-3	m2	DBA/2J	300	AML	134	c-Kit+/Gr-1+/Mac-1+	Bulk, 5e6	SAC	265	17.9	5.5	15.1	260	Unremarkable	40.6	Gr-1+/Mac-1+
m2-4	m2	DBA/2J	300	AML	134	c-Kit+/Gr-1+/Mac-1+	T-cell deplete bulk, 5e6	SAC	133	22.3	5.1	13.3	268	Mediastinal Mass	23.6	CD3+
m2-5	m2	DBA/2J	300	AML	134	c-Kit+/Gr-1+/Mac-1+	T-cell deplete bulk, 5e6	SAC	125	20.4	7.4	12.2	330	Splenomegaly	73.7	Lin-
m3-1	m3	DBA/2J	300	AML	159	c-Kit+/Gr-1+/Mac-1+	Bulk, 5e6	SAC	441	27.2	27.9	12.6	360	Splenomegaly, Kidney Mass	44.1	Gr-1+/Mac-1+
m3-2	m3	DBA/2J	300	AML	159	c-Kit+/Gr-1+/Mac-1+	Bulk, 5e6	SAC	446	26.3	5.2	11.1	235	Splenomegaly	76.2	Gr-1+/CD3+
m3-3	m3	DBA/2J	300	AML	159	c-Kit+/Gr-1+/Mac-1+	Bulk, 5e6	SAC	157	25.8	4.4	10.3	137	Splenomegaly, Adenopathy	98.9	c-Kit+/B220 lo
m3-4	m3	DBA/2J	300	AML	159	c-Kit+/Gr-1+/Mac-1+	T-cell deplete bulk, 1.6e6	EOE	427	31.5	7.7	10.1	296	Splenomegaly, Liver Tumor	23.9	B220+
m3-5	m3	DBA/2J	300	AML	159	c-Kit+/Gr-1+/Mac-1+	T-cell deplete bulk, 1.6e6	SAC	133	20.2	23.4	14.2	138	Unremarkable	19.4	B220 lo
m4-1	m4	DBA/2J	300	AML	160	Gr-1+/Mac-1+	Bulk, 4e6	SAC	265	29.3	5.8	12.1	269	Splenomegaly	96.7	Gr-1+/Mac-1+
m4-2	m4	DBA/2J	300	AML	160	Gr-1+/Mac-1+	Bulk, 4e6	SAC	265	28	2.4	9.8	355	Splenomegaly	88.7	B220 lo
m4-3	m4	DBA/2J	300	AML	160	Gr-1+/Mac-1+	Bulk, 4e6	SAC	265	27.8	13.4	11.9	453	Unremarkable	18.2	Gr-1+/Mac-1+
m4-4	m4	DBA/2J	300	AML	160	Gr-1+/Mac-1+	T-cell deplete bulk, 5e6	SAC	216	21.1	39.6	10.0	180	Splenomegaly, Mediastinal Mass	95.1	Lin-
m4-5	m4	DBA/2J	300	AML	160	Gr-1+/Mac-1+	T-cell deplete bulk, 5e6	FD	291	29.7	6.7	12.2	283	Splenomegaly	86.5	Gr-1+/Mac-1+
m5-1	m5	SWR/J	300	AML	215	c-Kit+/Gr-1+/Mac-1+	Bulk, 5e6	SAC	35	16.6	26.5	14.5	314	Splenomegaly	95.5	B220 lo
m5-2	m5	SWR/J	300	AML	215	c-Kit+/Gr-1+/Mac-1+	Bulk, 5e6	SAC	184	16.8	2.8	12.9	457	Unremarkable	27.9	CD3+
m5-3	m5	SWR/J	300	AML	215	c-Kit+/Gr-1+/Mac-1+	Bulk, 5e6	SAC	287	22.1	43.9	6.1	117	Splenomegaly	97.5	c-Kit+/Lin-
m5-4	m5	SWR/J	300	AML	215	c-Kit+/Gr-1+/Mac-1+	T-cell deplete bulk, 5e6	SAC	231	22.2	13.4	12.4	262	Splenomegaly	99.4	c-Kit+/Gr-1+/Mac-1+
m5-5	m5	SWR/J	300	AML	215	c-Kit+/Gr-1+/Mac-1+	T-cell deplete bulk, 5e6	SAC	309	21.6	12.2	11.0	169	Splenomegaly, Hepatic Infiltration	42.7	Lin-
m6-1	m6	DBA/2J	200	AML	182	c-Kit+/Gr-1+/Mac-1+	Bulk, 1e6	SAC	147	33.5	12.7	9.2	174	Splenomegaly	89.1	c-Kit+/CD3 lo
m6-2	m6	DBA/2J	200	AML	182	c-Kit+/Gr-1+/Mac-1+	Bulk, 1e6	SAC	291	34.2	19.7	7.8	345	Splenomegaly, Adenopathy	97.6	c-Kit+/Gr-1+/B220 lo
m6-3	m6	DBA/2J	200	AML	182	c-Kit+/Gr-1+/Mac-1+	Bulk, 1e6	SAC	221	29.7	5.9	10.3	205	Splenomegaly, Adenopathy	59.5	c-Kit+/Lin-
m7-1	m7	DBA/2J	200	AML	124	Gr-1+/Mac-1+	Bulk, 1e6	SAC	360	30.2	4.3	12.6	349	Splenomegaly	65.3	B220+
m7-2	m7	DBA/2J	200	AML	124	Gr-1+/Mac-1+	Bulk, 1e6	SAC	360	28.0	5.0	12.4	306	Splenomegaly	71.7	B220+
m7-3	m7	DBA/2J	200	AML	124	Gr-1+/Mac-1+	Bulk, 1e6	SAC	265	29.1	13.2	10.8	243	Splenomegaly	97.9	B220 lo
m8-1	m8	DBA/2J	100	AML	155	c-Kit+/Gr-1+/Mac-1+	Bulk, 1e6	SAC	16	20.3	5.5	11.4	97	Splenomegaly	71.7	c-Kit+/Gr-1+
m8-2	m8	DBA/2J	100	AML	155	c-Kit+/Gr-1+/Mac-1+	Bulk, 1e6	SAC	16	23.5	7.7	11.8	136	Splenomegaly	88.3	c-Kit+/Gr-1+
m8-3	m8	DBA/2J	100	AML	155	c-Kit+/Gr-1+/Mac-1+	Bulk, 1e6	SAC	16	19.2	8.5	12.2	136	Splenomegaly	91.4	c-Kit+/Gr-1+
m9-1	m9	DBA/2J	300	MDS	187	c-Kit+/Gr-1+/Mac-1+	Bulk, 5e6	SAC	348	19.7	1.7	10.7	619	Unremarkable	82.3	Gr-1+/Mac-1+
m9-2	m9	DBA/2J	300	MDS	187	c-Kit+/Gr-1+/Mac-1+	Bulk, 5e6	SAC	427	26.5	32.7	6.4	153	Splenomegaly	81.2	c-Kit+/B220 lo
m9-3	m9	DBA/2J	300	MDS	187	c-Kit+/Gr-1+/Mac-1+	Bulk, 5e6	SAC	383	24	4	15.1	365	Splenomegaly, Mediastinal Mass	82.4	B220+
m9-4	m9	DBA/2J	300	MDS	187	c-Kit+/Gr-1+/Mac-1+	T-cell deplete bulk, 5e6	SAC	308	22.7	9.7	5.9	490	Splenomegaly	10.4	B220+
m9-5	m9	DBA/2J	300	MDS	187	c-Kit+/Gr-1+/Mac-1+	T-cell deplete bulk, 5e6	SAC	255	17.8	38.2	12.1	482	Splenomegaly	28.0	c-Kit+/Gr-1+/Mac-1+
m10-1	m10	DBA/2J	300	MDS	134	Gr-1+/Mac-1+	Bulk, 4e6	SAC	357	23.5	1.1	5.8	108	Splenomegaly	97.0	Gr-1lo/Mac-1+/B220lo
m10-2	m10	DBA/2J	300	MDS	134	Gr-1+/Mac-1+	Bulk, 4e6	SAC	212	22.8	10	14.5	338	Splenomegaly	42.1	Gr-1+/CD3+
m10-3	m10	DBA/2J	300	MDS	134	Gr-1+/Mac-1+	Bulk, 4e6	SAC	229	27.4	2.7	6.3	312	Splenomegaly	90.8	Mac-1+
m10-4	m10	DBA/2J	300	MDS	134	Gr-1+/Mac-1+	T-cell deplete bulk, 2.3e6	SAC	327	23.5	2.4	12.2	410	Unremarkable	3.5	CD3+
m10-5	m10	DBA/2J	300	MDS	134	Gr-1+/Mac-1+	T-cell deplete bulk, 2.3e6	SAC	360	33.0	5.8	9.3	681	Splenomegaly, Intestine Tumor	25.7	B220 lo
m11-1	m11	DBA/2J	300	MDS	134	Gr-1+/Mac-1+	Bulk, 2.9e6	SAC	275	28.7	3.3	3.5	167	Splenomegaly	2.1	c-Kit+/Lin-
m11-2	m11	DBA/2J	300	MDS	134	Gr-1+/Mac-1+	Bulk, 2.9e6	SAC	275	29.9	4.3	5.7	105	Splenomegaly	11.1	c-Kit+/CD3 lo
m11-3	m11	DBA/2J	300	MDS	134	Gr-1+/Mac-1+	Bulk, 2.9e6	SAC	115	21.4	28.7	12.2	209	Splenomegaly, Mediastinal Mass	7.9	Gr-1+/Mac-1+
m11-4	m11	DBA/2J	300	MDS	134	Gr-1+/Mac-1+	T-cell deplete bulk, 2.4e6	FD	111	25.2	5.3	12.7	190	Unremarkable	0.7	CD3+
m11-5	m11	DBA/2J	300	MDS	134	Gr-1+/Mac-1+	T-cell deplete bulk, 2.4e6	SAC	273	28.3	9.1	5.8	66	Splenomegaly, Hepatomegaly	92.7	c-Kit+/Lin-
m12-1	m12	SWR/J	300	MDS	118	c-Kit+/Gr-1+/Mac-1+	Bulk, 5e6	SAC	183	27	16.6	9.3	263	Splenomegaly	89.9	c-Kit+/B220 lo
m12-2	m12	SWR/J	300	MDS	118	c-Kit+/Gr-1+/Mac-1+	Bulk, 5e6	SAC	155	19.8	3.8	12	318	Splenomegaly	87.0	c-Kit+/B220 lo
m12-3	m12	SWR/J	300	MDS	118	c-Kit+/Gr-1+/Mac-1+	Bulk, 5e6	SAC	92	29.5	5.1	6.1	169	Splenomegaly, Ovarian Tumor	91.1	Gr-1+/Mac-1+
m12-4	m12	SWR/J	300	MDS	118	c-Kit+/Gr-1+/Mac-1+	T-cell deplete bulk, 1.8e6	SAC	83	23.2	15.6	14.4	180	Unremarkable	8.3	CD3+
m12-5	m12	SWR/J	300	MDS	118	c-Kit+/Gr-1+/Mac-1+	T-cell deplete bulk, 1.8e6	SAC	116	23.0	37.5	12.9	322	Unremarkable	96.1	B220 lo
m13-1	m13	SWR/J	300	MDS	215	c-Kit+/Gr-1+/Mac-1+	Bulk, 4e6	SAC	422	20.9	9.6	9.2	307	Splenomegaly	58.3	B220+
m13-2	m13	SWR/J	300	MDS	215	c-Kit+/Gr-1+/Mac-1+	Bulk, 4e6	SAC	369	17.5	2.5	13.3	726	Splenomegaly	39.5	CD3+
m13-3	m13	SWR/J	300	MDS	215	c-Kit+/Gr-1+/Mac-1+	Bulk, 4e6	SAC	432	26.2	1.7	10.5	184	Splenomegaly, Hepatomegaly	56.4	CD3 lo
m13-4	m13	SWR/J	300	MDS	215	c-Kit+/Gr-1+/Mac-1+	T-cell deplete bulk, 4e6	SAC	290	20.1	5.0	12.1	342	Splenomegaly	75.1	CD3+
m13-5	m13	SWR/J	300	MDS	215	c-Kit+/Gr-1+/Mac-1+	T-cell deplete bulk, 4e6	SAC	301	29.5	105.5	7.9	268	Splenomegaly	1.5	Lin-
m14-1	m14	SWR/J	300	MDS	178	c-Kit+/Gr-1+	Bulk, 3e6	SAC	43	13.8	11	13.9	262	Splenomegaly	1.7	Gr-1+/Mac-1+
m14-2	m14	SWR/J	300	MDS	178	c-Kit+/Gr-1+	Bulk, 3e6	SAC	358	23.6	6.1	7.9	364	Splenomegaly	93.2	c-Kit+/Lin-
m14-3	m14	SWR/J	300	MDS	178	c-Kit+/Gr-1+	Bulk, 3e6	FD	46	22	2.8	11.1	271	Unremarkable	21.8	Gr-1+/Mac-1+
m14-4	m14	SWR/J	300	MDS	178	c-Kit+/Gr-1+	T-cell deplete bulk, 1.5e6	SAC	308	24.8	1.7	13.6	346	Unremarkable	1.3	CD3+
m14-5	m14	SWR/J	300	MDS	178	c-Kit+/Gr-1+	T-cell deplete bulk, 1.5e6	EOE	432	26.5	3.3	10.4	669	Unremarkable	19.2	CD3+
m15-1	m15	SWR/J	300	MDS	178	Gr-1+/Mac-1+	Bulk, 5e6	SAC	271	17.2	108.9	4.3	154	Splenomegaly	97.9	c-Kit+/B220 lo
m15-2	m15	SWR/J	300	MDS	178	Gr-1+/Mac-1+	Bulk, 5e6	SAC	143	20.2	3.1	12.5	263	Splenomegaly	21.4	c-Kit+/Gr-1+/Mac-1+
m15-3	m15	SWR/J	300	MDS	178	Gr-1+/Mac-1+	Bulk, 5e6	SAC	293	21.4	5.7	8.7	303	Splenomegaly, Vertebral Mass	87.4	Mac-1+/B220 lo
m15-4	m15	SWR/J	300	MDS	178	Gr-1+/Mac-1+	T-cell deplete bulk, 1.5e6	EOE	308	30.5	2.1	12.8	373	Adenopathy	0.2	Mac-1+
m15-5	m15	SWR/J	300	MDS	178	Gr-1+/Mac-1+	T-cell deplete bulk, 1.5e6	EOE	308	29.2	2.6	11.6	338	Liver Tumor	0.2	CD3+
m16-1	m16	DBA/2J	100	MDS	262	Gr-1+/Mac-1+	Bulk, 1e6	EOE	275	25.6	2.9	14.3	266	Unremarkable	82.6	B220+
m16-2	m16	DBA/2J	100	MDS	262	Gr-1+/Mac-1+	Bulk, 1e6	EOE	275	27.9	2.4	13.3	302	Unremarkable	59.7	B220+
m16-3	m16	DBA/2J	100	MDS	262	Gr-1+/Mac-1+	Bulk, 1e6	EOE	275	25.9	5.5	13.8	310	Unremarkable	73.4	B220+
m17-1	m16	DBA/2J	300	MDS	182	c-Kit+/Gr-1+/Mac-1+	Bulk, 1e6	SAC	82	32.8	8.0	12.3	333	Eye Inflammation	22.9	B220+
m17-2	m16	DBA/2J	300	MDS	182	c-Kit+/Gr-1+/Mac-1+	Bulk, 1e6	SAC	167	29.1	12.7	10.0	586	Splenomegaly, Hepatomegaly	4.7	CD3+
m17-3	m16	DBA/2J	300	MDS	182	c-Kit+/Gr-1+/Mac-1+	Bulk, 1e6	SAC	138	26.6	6.0	12.8	187	Unremarkable	16.1	CD3+

SAC = Sacrificed for moribund status
FD = Found dead
EOE = Sacrificed at end of experiment
ND = No data
Wt/CBC values are the last recorded, usually at sacrifice
Splenomegaly defined as > 1.5cm in length
Engraftment values are % mCD45.2+ cells and are the last recorded, usually at sacrifice

Table C in S1 File. Characteristics of mice transplanted with human t-AML samples (SU108, SU158 and SU190).

SU ID	Donor Cell Type, Dose	Mouse Fate	Survival After Transplant	Weight	WBC	Hgb	Plt	Necropsy	PB Engraftment	BM Engraftment	Spleen Engraftment
SU108	CD3 depleted bulk cells, 4e6	SAC	67	21.2	0.6	6.7	69	Splenomegaly, Hepatic infiltration	54.0	97.5	97.4
SU108	CD3 depleted bulk cells, 4e6	SAC	60	22.9	0.7	6.1	66	Splenomegaly	34.9	94.4	95.3
SU108	CD3 depleted bulk cells, 4e6	SAC	67	20.5	0.8	7.1	73	Splenomegaly, Hepatic infiltration	73.8	97.9	98.5
SU108	CD3 depleted bulk cells, 4e6	SAC	67	21.6	0.8	5.8	60	Splenomegaly, Hepatic infiltration	56.6	98.2	98.1
SU108	CD3 depleted bulk cells, 4e6	FD	64	23.4	0.6	10.1	183	ND	21.1	ND	ND
SU108	CD34-, 100,000	EOE	218	26.0	2.9	13.3	248	Unremarkable	0.3	0.0	0.3
SU108	CD34-, 100,000	EOE	218	26.2	2.1	13.1	200	Unremarkable	0.9	0.2	0.3
SU108	CD34-, 100,000	EOE	218	29.9	3.6	12.8	328	Unremarkable	0.4	0.7	0.5
SU108	CD34+/CD38+, 100,000	FD	102	26.3	1.0	12.3	232	ND	0.4	ND	ND
SU108	CD34+/CD38+, 100,000	EOE	218	29.8	4.4	12.8	379	Unremarkable	0.4	0.0	0.5
SU108	CD34+/CD38+, 100,000	SAC	141	ND	1.1	6.3	136	Splenomegaly, Hepatic infiltration	19.5	42.1	96.0
SU108	CD34+/CD38-, 100,000	SAC	111	25.1	0.5	8.3	99	Splenomegaly	79.5	71.6	98.0
SU108	CD34+/CD38-, 100,000	SAC	111	22.8	1.9	4.7	94	Splenomegaly, Adenopathy	53.3	78.9	87.5
SU108	CD34+/CD38-, 100,000	SAC	102	21.6	1.1	5.4	91	Splenomegaly, Hepatic infiltration	89.9	36.8	99.5
SU108	CD34+/CD38-, 250,000	FD	72	26.1	1.1	11.7	195	Splenomegaly	5.9	ND	ND
SU158	CD3 depleted bulk cells, 2.5e6	SAC	81	17.1	1.9	11.7	171	Mild Adenopathy	96.9	99.7	85.5
SU158	CD3 depleted bulk cells, 2.5e6	SAC	127	26.6	2.4	9.5	289	Unremarkable	64.6	95.6	65.2
SU158	CD3 depleted bulk cells, 2.5e6	SAC	116	27.1	2.4	6.6	246	Splenomegaly, Adenopathy	98.7	94.1	96.0
SU158	CD3 depleted bulk cells, 2.5e6	SAC	120	17.2	10.0	17.1	234	Adenopathy	71.3	94.7	96.1
SU158	CD3 depleted bulk cells, 2.5e6	SAC	81	18.0	3.0	8.2	137	Unremarkable	99.1	99.6	82.1
SU158	CD34-, 100,000	EOE	217	35.2	1.3	11.4	383	Unremarkable	23.9	99.4	19.0
SU158	CD34-, 100,000	EOE	217	32.1	1.2	11.6	429	Unremarkable	19.4	98.0	9.4
SU158	CD34-, 100,000	EOE	217	37.5	1.3	10.5	158	Unremarkable	74.1	97.3	55.2
SU158	CD34+/CD38+, 100,000	EOE	217	30.4	1.0	10.9	215	Unremarkable	31.1	99.3	15.5
SU158	CD34+/CD38+, 100,000	SAC	214	21.8	2.7	7.9	294	Unremarkable	95.0	92.5	97.3
SU158	CD34+/CD38+, 100,000	EOE	217	27.1	1.6	11.6	270	Unremarkable	63.4	99.7	31.6
SU158	CD34+/CD38-, 100,000	SAC	178	22.0	3.5	12.4	221	Adenopathy	36.7	82.0	24.6
SU158	CD34+/CD38-, 100,000	EOE	217	30.2	1.1	12.0	332	Unremarkable	3.8	71.7	1.5
SU158	CD34+/CD38-, 100,000	SAC	178	23.7	1.5	3.7	121	Unremarkable	96.5	94.6	97.9
SU158	CD34+/CD38-, 250,000	SAC	124	23.0	0.6	7.9	152	Unremarkable	83.1	86.8	82.8
SU190	CD3 depleted bulk cells, 5e6	SAC	31	15.6	5.0	11.4	99	Splenomegaly, Adenopathy	60.1	92.0	84.1
SU190	CD3 depleted bulk cells, 5e6	SAC	28	15.8	1.9	11.9	206	Splenomegaly	52.6	86.6	80.5
SU190	CD3 depleted bulk cells, 5e6	SAC	31	17.9	3.4	11.0	163	Splenomegaly	40.6	95.4	78.4
SU190	CD3 depleted bulk cells, 5e6	SAC	31	17.9	2.1	10.2	183	Splenomegaly	41.1	86.2	77.5
SU190	CD3 depleted bulk cells, 5e6	SAC	31	18.1	1.8	9.4	144	Splenomegaly	75.3	98.9	83.2
SU190	CD34-, 10,000	EOE	225	27.6	3.0	13.5	295	Unremarkable	0.5	0.1	0.3
SU190	CD34-, 10,000	SAC	48	20.0	4.0	12.4	201	Splenomegaly	91.0	87.0	96.6
SU190	CD34-, 10,000	SAC	158	23.7	6.2	13.3	277	Splenomegaly	52.9	80.6	90.9
SU190	CD34+/CD38+, 10000	EOE	225	35.1	1.0	12.6	399	Unremarkable	5.4	93.3	7.1
SU190	CD34+/CD38+, 10000	EOE	225	28.6	5.5	11.4	358	Unremarkable	8.4	97.7	9.5
SU190	CD34+/CD38+, 10000	EOE	225	33.3	1.6	12.3	353	Unremarkable	7.4	94.1	7.6
SU190	CD34+/CD38-, 10000	EOE	225	31.6	0.4	9.9	342	Unremarkable	18.4	99.5	29.8
SU190	CD34+/CD38-, 10000	EOE	225	33.2	0.4	11.1	127	Unremarkable	19.5	99.4	26.7
SU190	CD34+/CD38-, 10000	EOE	225	29.3	1.4	8.7	119	Unremarkable	44.3	99.4	31.2
MOCK	PBS	EOE	225	34.9	2.9	13.0	203	Unremarkable	0.3	0.2	0.3

SAC = Sacrificed for moribund status
FD = Found dead
EOE = Sacrificed at end of experiment
ND = No data
Wt/CBC values are the last recorded, usually at sacrifice
Splenomegaly defined as > 1.5cm in length
Engraftment values are % hCD45+/CD33+ cells and are the last recorded, usually at sacrifice
